# A force-sensitive mutation reveals a spindle assembly checkpoint-independent role for dynein in anaphase progression

**DOI:** 10.1101/2023.08.03.551815

**Published:** 2023-08-04

**Authors:** David Salvador-Garcia, Li Jin, Andrew Hensley, Mert Gölcük, Emmanuel Gallaud, Sami Chaaban, Fillip Port, Alessio Vagnoni, Vicente José Planelles-Herrero, Mark A. McClintock, Emmanuel Derivery, Andrew P. Carter, Régis Giet, Mert Gür, Ahmet Yildiz, Simon L. Bullock

**Affiliations:** 1 Cell Biology Division, MRC Laboratory of Molecular Biology, Cambridge, CB2 0QH, UK; 2 Department of Physics, University of California at Berkeley, Berkeley, CA 94720, USA; 3 Department of Mechanical Engineering, Istanbul Technical University, Istanbul, 34437, Turkey; 4 Institut de Génétique et Développement de Rennes - UMR 6290, Université de Rennes, F-35000 Rennes, France; 5 Structural Studies Division, MRC Laboratory of Molecular Biology, Cambridge, CB2 0QH, UK; 6 Current address: Division of Signaling and Functional Genomics, German Cancer Research Center (DKFZ), 69120 Heidelberg, Germany; 7 Current address: Department of Basic and Clinical Neuroscience, Institute of Psychiatry, Psychology and Neuroscience, King’s College London, London, SE5 9RX, UK; 8 Department of Computational and Systems Biology, University of Pittsburgh, Pittsburgh, PA 15260, USA; 9 Department of Molecular and Cellular Biology, University of California at Berkeley, Berkeley, CA 94720, USA

**Keywords:** dynein, mitosis, *Drosophila*, microtubule-binding domain, force production, kinetochore, axonal transport

## Abstract

The cytoplasmic dynein-1 (dynein) motor organizes cells by shaping microtubule networks and moving a large variety of cargoes along them. However, dynein’s diverse roles complicate *in vivo* studies of its functions significantly. To address this issue, we have used gene editing to generate a series of missense mutations in *Drosophila* Dynein heavy chain (Dhc). We find that mutations associated with human neurological disease cause a range of defects in larval and adult flies, including impaired cargo trafficking in neurons. We also describe a novel mutation in the microtubule-binding domain (MTBD) of Dhc that, remarkably, causes metaphase arrest of mitotic spindles in the embryo but does not impair other dynein-dependent processes. We demonstrate that the mitotic arrest is independent of dynein’s well-established roles in silencing the spindle assembly checkpoint. *In vitro* reconstitution and optical trapping assays reveal that the mutation only impairs the performance of dynein under load. *In silico* all-atom molecular dynamics simulations show that this effect correlates with increased flexibility of the MTBD, as well as an altered orientation of the stalk domain, with respect to the microtubule. Collectively, our data point to a novel role of dynein in anaphase progression that depends on the motor operating in a specific load regime. More broadly, our work illustrates how cytoskeletal transport processes can be dissected *in vivo* by manipulating mechanical properties of motors.

## INTRODUCTION

Microtubule motors play a key role in organizing the intracellular environment. These molecules power the movement of a wide variety of cellular constituents along microtubules (Hirokawa et al, 2009; Reck-Peterson et al, 2018) and provide pushing and pulling forces that shape microtubule networks (Cross & McAinsh, 2014; Laan et al, 2012; Lu & Gelfand, 2017).

**Figure 1.**
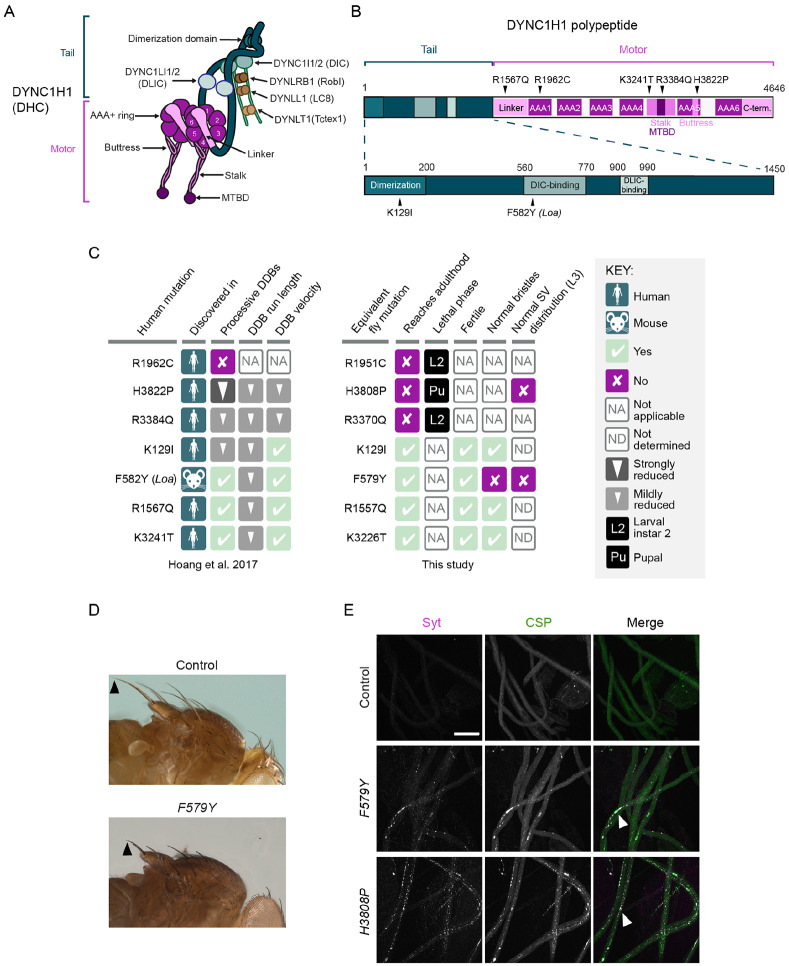
Dynein organization and phenotypic analysis of Dhc disease-associated mutations in *Drosophila*. (A) Cartoon of human dynein complex with alternative nomenclature for subunits shown. MTBD, microtubule-binding domain. The C-terminal domain of DYNC1H1 is not visible in this view as it lies on the other face of the AAA+ rings. (B) Positions in the human DYNC1H1 polypeptide of the disease-associated mutations characterized in this study. Subdomains of the DYNC1H1 polypeptide are color-coded as in panel A. The mouse *Loa* mutation is numbered according to the equivalent residue in human DYNC1H1. Adapted from Hoang et al, 2017. (C) Summary of *in vitro* and *in vivo* effects of disease-associated mutations. DDB, dynein-dynactin-BICD2N; SV, synaptic vesicle; L3, larval instar 3. *In vitro* effects refer to observations when both copies of DYNC1H1 in the dynein complex contain the mutation; *in vivo* phenotypes refer to the homozygous condition. (D) Images showing short bristles on the notum of homozygous *Dhc*^*F579Y*^ adult flies compared to controls (*yw*). Arrowheads point to posterior scutellar macrochaetae as an example. The bristle phenotype of *Dhc*^*F579Y*^ flies was completely penetrant (>160 flies examined). (E) Confocal images of segmental nerves (taken proximal to the ventral ganglion; anterior to the top; Z-projections) from fixed L3 larvae stained for the synaptic vesicle proteins Synaptotagmin (Syt) and Cysteine-string protein (CSP). Arrowheads show examples of synaptic vesicle accumulations in mutants. Images are representative of 3 – 6 larvae analyzed per genotype. Scale bar: E, 50 μm.

The cytoplasmic dynein-1 (dynein) motor is responsible for almost all microtubule minus-end-directed motor activity in the cytoplasm. Dynein has six subunits – a heavy chain, an intermediate chain, a light intermediate chain and three light chains – which are each present in two copies per complex (Figure 1A). The heavy chain has over 4600 amino acids and comprises an N-terminal tail domain, which mediates homodimerization and association with the other subunits, and a C-terminal motor domain. The key elements of the motor domain are a ring of six AAA+ ATPase domains, a linker, and an anti-parallel coiled-coil stalk that leads to a globular microtubule-binding domain (MTBD) (Figure 1A; Schmidt & Carter, 2016). The major ATP hydrolysis site in dynein is located in the first AAA+ domain (AAA1). ATP binding at this site triggers conformational changes in the ring that are transmitted, via a shift in the stalk’s coiled-coil registry, to the MTBD. This event lowers the affinity of the MTBD for the microtubule, leading to its detachment. ATP hydrolysis and product release allow the MTBD to rebind the microtubule, initiating a swing of the linker that produces force. Repeated cycles of these events drive the movement of dynein along the microtubule. Dynein’s force output and processivity are greatly stimulated by simultaneous binding of two co-factors to the tail domain: another large, multi-subunit complex called dynactin, and a long, coiled-coil-containing cargo adaptor, which is termed an activating adaptor (Belyy et al, 2016; McKenney et al, 2014; Olenick & Holzbaur, 2019; Reck-Peterson et al, 2018; Schlager et al, 2014).

The wide variety of dynein functions in cells makes it very challenging to assess the function of the motor in discrete processes. Mutations that strongly disrupt dynein function are typically not compatible with survival (Gepner et al, 1996; Harada et al, 1998; Mische et al, 2008) and acute inhibition of the motor complex with function-blocking antibodies (e.g. Gaglio et al, 1997; Sharp et al, 2000; Yang et al, 2007; Yi et al, 2011) or small molecule inhibitors (e.g. Firestone et al, 2012; Hoing et al, 2018; Steinman et al, 2017) impairs multiple processes that can be difficult to disentangle.

A striking illustration of the complexity of studying dynein function is in mitosis. Here, the motor has been implicated in centrosome separation (Gonczy et al, 1999; Raaijmakers et al, 2012; Robinson et al, 1999; Vaisberg et al, 1993; van Heesbeen et al, 2014), nuclear envelope breakdown (Salina et al, 2002), spindle pole focusing (Borgal & Wakefield, 2018; Gaglio et al, 1997; Merdes et al, 1996), attachment of kinetochores to microtubules and chromosome congression (Barisic & Maiato, 2015; Gassmann et al, 2008; Li et al, 2007; Varma et al, 2008; Yang et al, 2007), creating tension between sister kinetochores (Sivaram et al, 2009; Varma et al, 2008; Yang et al, 2007), and silencing the spindle assembly checkpoint (SAC) by stripping regulatory factors from kinetochores (Gassmann et al, 2010; Griffis et al, 2007; Hinchcliffe & Vaughan, 2017; Howell et al, 2001; Sivaram et al, 2009; Wojcik et al, 2001). It was proposed that dynein is also directly responsible for segregating chromosomes in anaphase (Savoian et al, 2000; Sharp et al, 2000; Yang et al, 2007). However, other proteins have since been shown to be essential for poleward chromosome movement and it has been suggested that delays in this process when dynein is inhibited arise indirectly from perturbing the motor’s other functions at the kinetochore (Bader & Vaughan, 2010; Maddox et al, 2002; Vukusic et al, 2019).

In this study, we have taken a combined genetic and biochemical approach to dissect dynein’s diverse functions. We first document organismal and cellular effects of a large number of Dynein heavy chain missense mutations – including several that are associated with human neurological disease – in the fruit fly, *Drosophila melanogaster*. We then focus on a novel mutation in the MTBD – S3372C – that specifically blocks mitosis in the early embryo. We show that this mutation prevents progression of mitotic spindles from metaphase independently of dynein’s well-established function in licensing anaphase onset by silencing the SAC. High-resolution optical trapping of *in vitro* reconstituted dynein-dynactin-activating adaptor complexes reveals that the MTBD mutation only impairs dynein performance when it encounters resistive loads. All-atom molecular dynamics (MD) simulations show that the S3372C change causes dynein to bind to the microtubule with enhanced flexibility and an altered relative orientation of the stalk. Based on these observations, we propose that dynein has a non-canonical function in anaphase progression that involves the motor operating in a specific force regime. Our work illustrates the feasibility of dissecting the function of cytoskeletal motors by interfering with specific mechanical properties.

## RESULTS

### Characterization of disease-associated Dhc mutations in *Drosophila*

We first set out to study the *in vivo* effects of six neurological-disease-linked missense mutations in the human Dynein heavy chain protein (DYNC1H1): K129I, R1567Q, R1962C, 3384Q, K3241T and H3822P (Figure 1B). Heterozygosity for each of these mutations is associated with malformations in cortical development and intellectual disability (Poirier et al, 2013; Schiavo et al, 2013; Vissers et al, 2010; Willemsen et al, 2012). We previously used *in vitro* reconstitution to show that these mutations perturb, to varying degrees, the processive movement of human dynein complexes bound to dynactin and the N-terminal region of the prototypical activating adaptor, BICD2 (so-called ‘DDB’ complexes) (Hoang et al, 2017; results summarized in Figure 1C). Whether these inhibitory effects accounted fully for the *in vivo* consequences of these mutations was unclear.

We therefore used CRISPR/Cas9-based homology-directed repair (HDR) to generate *Drosophila* strains with equivalent mutations in the gene encoding Dynein heavy chain (*Dhc64C*, hereafter *Dhc*) (Figure 1C). We also made a strain carrying the *Drosophila* equivalent of the human F582Y mutation (F579Y), which corresponds to the *Legs-at-odd-angles* (*Loa*) allele that causes neurodegenerative disease in heterozygous mice (Hafezparast et al, 2003). This mutation also impairs the movement of DDB complexes along microtubules *in vitro* (Hoang et al, 2017; Figure 1C).

None of the mutations caused overt phenotypes when heterozygous in flies. However, animals homozygous for three of the mutations (R1962C human/R1951C fly; H3822P human/H3808P fly; R3384Q human/R3370Q fly) failed to reach adulthood (Figure 1C). Complementation tests with a *Dhc* protein null allele (Fumagalli et al, 2021) confirmed that lethality was due to the missense mutations rather than off-target activity of CRISPR/Cas9 (Figure S1A). Whereas homozygous *Dhc* protein null mutants died during both early and late phases of the second larval instar stage (L2), the vast majority of *R1951C* and *R3370Q* mutants arrested during early L2 (Figure 1C and Figure S1B). The slightly earlier lethal phase of R1951C and R3370Q compared to the *Dhc* null suggests that these mutations have a partially dominant negative effect. *H3808P* mutants typically died during the pupal phase (Figure 1C and Figure S1B), demonstrating that this mutation retains some dynein activity *in vivo*. Of the four homozygous viable mutations (K129I, F579Y, R1557Q (equivalent to R1567Q) and K3226T (equivalent to human K3241T)), only F579Y caused a morphological defect in adults. Homozygotes for this mutation had abnormally short bristles on the notum (Figure 1D), which is a feature of several classical hypomorphic *Dhc* mutations (Gepner et al, 1996; Melkov et al, 2016).

We previously proposed that the pathomechanism of the disease-associated mutations involves impaired trafficking of cargo-motor complexes in neurons (Hoang et al, 2017). To investigate this notion, we examined the effects of the H3808P and F579Y mutations on the distribution of synaptic vesicles in axons of segmental nerve motor neurons in larval instar 3(L3). Synaptic vesicles in this system undergo dynein- and kinesin-1-dependent bidirectional transport (Miller et al, 2005), and impairing the activity of either motor causes focal accumulations of the vesicles that are visible by immunofluorescence (Martin et al, 1999). Homozygosity for the *H3808P* or *F579Y* alleles caused striking synaptic vesicle accumulations in axons (Figure 1E), supporting the hypothesis that disease-associated mutations interfere with cargo translocation in neurons.

Figure 1C summarizes the effects of the disease-associated mutations in *Drosophila* and on DDB activity *in vitro* (Hoang et al, 2017). The three mutations that had the strongest effects on DDB behavior *in vitro* – R1962C, R3384Q, and H3822P – were those whose counterparts caused lethality in flies. However, the relative strengths of these mutations’ *in vitro* and *in vivo* effects were not equivalent. Even though H3822P impaired *in vitro* motility of DDB more strongly than R3384Q, the equivalent mutation resulted in later lethality in flies than the R3384Q equivalent. The milder phenotype of the H-to-P mutation in *Drosophila* was particularly surprising as this change strongly disrupts dynein function in budding yeast (Marzo et al, 2019). Moreover, whereas the effect of the human F582Y mutation on DDB *in vitro* motility was very similar to that of R1567Q and K3241T, and weaker than that of K129I, the equivalent *Drosophila* mutation (F579Y) was unique amongst this group in disrupting bristle morphology. These observations might reflect some mutations having species specific effects or altering dynein’s interplay with proteins in addition to dynactin and the BICD2 orthologue, BicD. Nonetheless, the range of mutant phenotypes observed in *Drosophila* and the overall correlation with the strength of inhibitory effects *in vitro* indicate that the fly is a valuable model for structure-function studies of the dynein complex.

### The novel mutation S3372C specifically impairs a function of dynein during embryonic development

Whilst generating the disease-associated mutations in *Drosophila* we recovered four novel in-frame Dhc mutations that were caused by imprecise repair of CRISPR/Cas9-induced double strand breaks. L1952K and the two-amino-acid deletion ΔL3810Y3811, which came from the experiments that produced R1951C and H3808P, respectively, caused lethality in homozygotes (Figure S2A) or when combined with the null allele (Figure S1A). Homozygosity for each of these mutations resulted in arrest during L3 and accumulations of synaptic vesicles in motor neuron axons (Figure S2A and B). Y3811F was also recovered whilst making H3808P, although the homozygous mutants did not have a detectable phenotype (Figures S1A and S2A).

**Figure 2.**
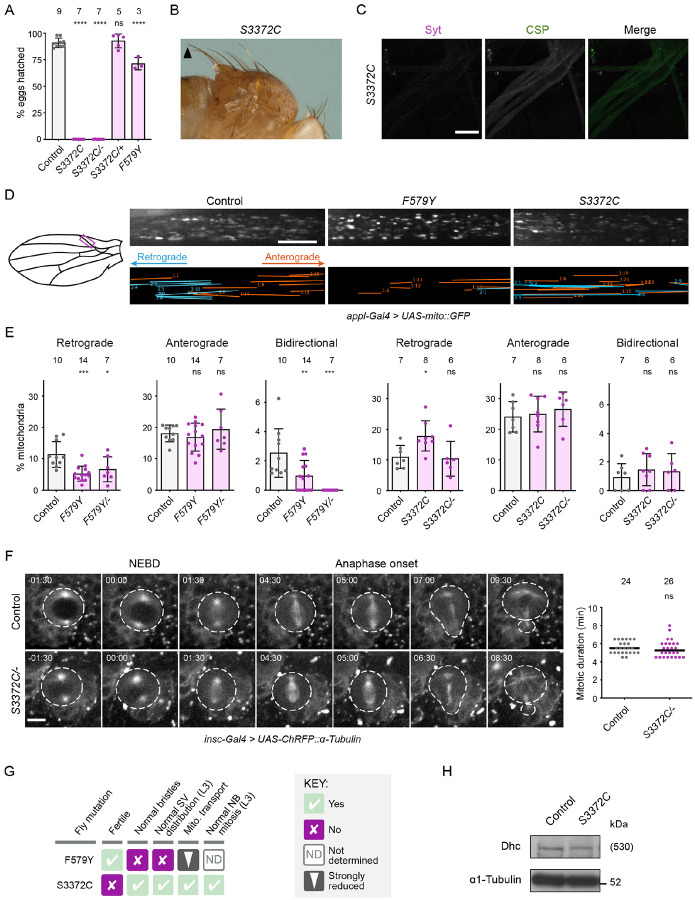
The novel mutation S3372C selectively affects embryonic development. (A) Hatching rate of eggs laid by mated females of the indicated genotypes. Columns show mean values per egg collection; error bars represent S.D.; circles are values for individual egg collections. Number of collections per genotype (from 3 independent crosses) is shown above columns (114 – 575 eggs per collection). *S3372C/-* are *trans*-heterozygous for *S3372C* and a *Dhc* null allele. Control genotype: *yw*. (B, C) Images showing (B) normal bristle length in adults and (C) lack of synaptic vesicle accumulations in segmental nerves of L3 larvae (proximal to the ventral ganglion; anterior to the top; Z-projection) in *S3372C* homozygotes (for comparisons with controls, see Figure 1D and E). Arrowhead in B points to posterior scutellar macrochaetae. Images in B and C are representative of >160 flies and 3 larvae analyzed, respectively. (D) Analysis of mitochondrial motility in wing nerve axons of 2-day-old wild-type, *F579Y* and *S3372C* adult flies. Left, cartoon of wing with magenta box indicating region imaged. Top images, example stills from 3-minute time series (single focal plane) of fluorescent mitochondria (expression of mito::GFP in neurons using *Appl-Gal4*). Bottom images, example traces of motile mitochondria in corresponding time series. Blue, retrograde tracks; orange, anterograde tracks. (E) Percentages of mitochondria transported in the retrograde or anterograde directions, or moving bidirectionally during the 3 minutes of data acquisition. Columns show mean values per movie; error bars represent S.D.; circles are values for individual movies (each from a different wing). Number of wings analyzed shown above bars. Note that we observed an increased frequency of retrograde transport in *S3372C* homozygotes but this was not recapitulated in *S3372C/-* animals. (F) Analysis of mitotic duration in neuroblasts (NBs) in the brain of control and *S3372C/-* mutant L3 larvae. Left, stills from image series of NBs with fluorescently-labeled spindles (expression of RFP-tagged α-tubulin in NBs using *insc-Gal4*). NBs and daughter cells highlighted with dashed line. Timestamps are min:s after nuclear envelope breakdown (NEBD). Right, quantification of mitotic duration (NEBD to anaphase onset). Lines show median; circles are values for individual NBs. Numbers of neuroblasts analyzed (from 4 wild-type or 5 *S3372C/-* larvae) is shown above plot. (G) Summary of *in vivo* effects of homozygosity for *F579Y* and *S3372C*. SV, synaptic vesicle; Mito., mitochondrial. (H) Immunoblot of extracts of embryos from control and *S3372C* mothers (0 – 160 min collections), probed with antibodies to Dhc and α1-tubulin (loading control). Position of molecular weight (Mw) marker is shown for α1-tubulin blot segment; as there is no marker where Dhc migrates, the predicted Mw of Dhc is shown in parentheses. Evaluation of statistical significance (compared to control) was performed with a 1-way ANOVA with Dunnett’s multiple comparisons tests (A and E) or a Mann-Whitney test (F): ****, *P*<0.0001; ***, P<0.001; **, P<0.01; *, P<0.05; ns, not significant. Scale bars: C, 50 μm; D, 10 μm; F, 5 μm.

The fourth novel mutation, S3372C, was a by-product of our efforts to make R3370Q. This mutation, which is situated in the MTBD, had a particularly striking phenotype. Whilst *S3372C* homozygous adults were recovered at the normal Mendelian frequency and males were fertile, homozygous females had very strongly impaired fertility (0.029% of eggs hatched into larvae; Figure 2A). No fertility problems were observed in females heterozygous for the *S3372C* allele, showing that this is a recessive effect. Like *S3372C* homozygotes, *trans*-heterozygotes for the *S3372C* allele and the *Dhc* protein null allele (*S3372C/-*) had normal survival to adulthood (Figure S1A) and female-specific infertility (Figure 2A). Thus, the female fertility defect is associated with the S3372C mutation rather than an off-target effect of CRISPR/Cas9. This conclusion was corroborated by the ability of a wild-type *Dhc* genomic construct to restore fertility to *S3372C* homozygotes (Figure S2C).

In contrast to S3372C, the non-lethal disease-associated *Dhc* mutations did not strongly affect fertility (Figure 1C). For example, only 30% of embryos from *F579Y* homozygous mothers failed to hatch (Figure 2A). Moreover, despite having much stronger fertility defects, *S3372C* homozygous and *S3372C/-* animals did not have the bristle defects (Figure 2B and Figure S2D) or focal accumulations of synaptic vesicles (Figure 2C and Figure S2E) observed with the F579Y mutation. These observations reveal that S3372C only affects a subset of dynein functions.

To further explore which dynein-dependent processes are affected by S3372C, we used time-lapse imaging to compare trafficking of GFP-labeled mitochondria in wing nerve axons of adult wild-type and mutant flies (Vagnoni et al, 2016). In these axons, as in others, dynein and kinesin-1 are responsible for retrograde and anterograde mitochondrial movements, respectively (Vagnoni et al, 2016). The F579Y mutation was used as a positive control for the experiments, as the equivalent mutation impairs dynein-based cargo transport in other organisms (Hafezparast et al, 2003; Ori-McKenney et al, 2010; Sivagurunathan et al, 2012). Homozygosity for *F579Y* allele reduced the frequency of retrograde and bidirectional transport events of mitochondria in wing nerve axons without impairing retrograde velocity or travel distance, or any aspect of anterograde motion (Figure 2D and E and Figure S2F and G). In contrast, the S3372C mutation did not significantly impair any aspect of retrograde or anterograde motion of mitochondria (Figure 2D and E and Figure S2F and G). We also assessed the effect of S3372C on cell division of neuroblasts of live L3 brains expressing fluorescent α-tubulin. Inhibiting dynein and dynactin function in these neuroblasts perturbs spindle morphology and delays the completion of mitosis (Siller et al, 2005; Wojcik et al, 2001). In *S3372C/-* mutant neuroblasts, however, no spindle abnormalities were observed and the duration of mitosis was indistinguishable from the wild type (Figure 2F). Thus, the S3372C mutation has no discernible inhibitory effect on dynein functions in axonal transport of mitochondria or mitosis of larval neuroblasts.

Taken together, our phenotypic analyses (summarized in Figure 2G) reveal a selective maternal effect of S3372C on dynein function during embryogenesis. A trivial reason for this observation could be that the mutation destabilizes Dhc protein in the embryo. However, immunoblotting of wild-type and *S3372C* embryo extracts with an α-Dhc antibody showed that this is not the case (Figure 2H). Therefore, the MTBD mutation affects the functionality, rather than the level, of Dhc in embryos.

### The conserved location of S3372 indicates an important role in MTBD function

**Figure 3.**
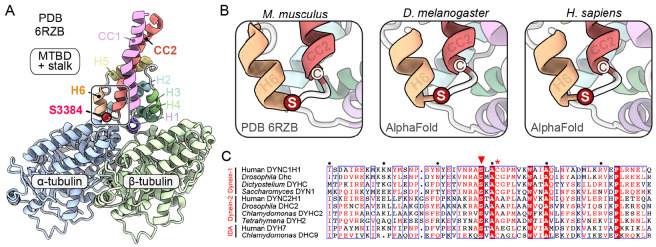
S3372 has a conserved position in a loop between H6 of the MTBD and CC2 of the stalk. (A) Overview of position of the equivalent residue to S3372 (S3384) in the cryo-EM structure of the mouse MTBD and portion of the stalk in complex with the α/β-tubulin dimer (PDB, 6RZB). Position of S3384 is highlighted by a red circle. (B) Zoom ins of regions containing S3384 in 6RZB and equivalent residues (red circles) in Alphafold2-generated structures of the MTBD and stalk of *Drosophila melanogaster* Dhc and human DYNC1H1. Neighbouring cysteine residues (C3387 mouse; C3375 *Drosophila*; C3389 human) are also shown. (C) Alignment of sequences from the MTBD and CC2 of the stalk of the indicated dynein family members. White letters on a red background, residues present in all sequences; red letters, residues present in ≥ 50% of sequences; blue boxes, regions with ≥ 50% conservation; red arrowhead, residues equivalent to S3372 of *Drosophila* Dhc; red asterisk, residues equivalent to C3375 of *Drosophila* Dhc. Uniprot accession numbers: human (*Homo sapiens*) DYNC1H1, Q14204; *Drosophila melanogaster* Dhc, P37276; *Dictyostelium discoideum* DYHC, P34036; *Saccharomyces cerevisiae* DYN1, P36022; human (*Homo sapiens*) DYNC2H1, Q8NCM8; *Drosophila melanogaster* DHC2, Q0E8P6; *Chlamydomonas reinhartii* DYHC2, Q9SMH5; *Tetrahymena thermophila* DYH2, Q5U9X1; human (*Homo sapiens*) inner dynein arm (IDA) DYH7, Q8WXX0; *Chlamydomonas reinhartii* inner dynein arm (IDA) DHC9, Q4AC22.

To attempt to gain insight into how mutating S3372 affects dynein activity, we examined its position within the tertiary structure of the MTBD. Whilst an experimentally determined structure of the *Drosophila* dynein MTBD is not available, we could confidently infer the position of the serine from a 4.1 Å-resolution cryo-electron microscopy (EM) structure of the closely related mouse MTBD, together with a portion of the stalk, bound to an α/β-tubulin dimer in the microtubule (Figure 3A; Lacey et al, 2019). The equivalent residue in mouse (S3384) is located in a short loop that connects helix 6 (H6) of the MTBD – which directly contacts α-tubulin – to the base of coiled-coil 2 (CC2) of the stalk (Figure 3A and B). We also used Alphafold2 (Jumper et al, 2021) to produce high-confidence structural predictions of the MTBD and stalk of the *Drosophila* and human dynein-1 heavy chains (Figure 3B and Figure S3A). In both these structures, the serine adopted an equivalent position to that observed in the cryo-EM structure of the mouse MTBD.

The conserved position of the serine in dynein-1 from fly, mouse and human suggests that it plays an important role in these contexts. To determine if this residue could have broader significance, we examined its conservation in other dynein-1 proteins, as well as in axonemal dyneins and dynein-2, which drive the beating of cilia and movement of material within these structures, respectively (Antony et al, 2021). The serine was conserved in the primary sequence of all dynein-1, dynein-2, and axonemal dynein heavy chain sequences examined, including those from single cell eukaryotes (Figure 3C). Alphafold2-generated predictions of MTBD and stalk regions from the human dynein-2 heavy chain and axonemal DYH7 proteins (Figure S3A) indicated that the location of the serine in a loop between H6 and CC2 is also conserved (Figure S3B). These analyses suggest that the serine that is mutated in the *S3372C* fly strain plays an important role across the dynein family.

### Ectopic disulfide bond formation does not account for the S3372C phenotype

**Figure 4.**
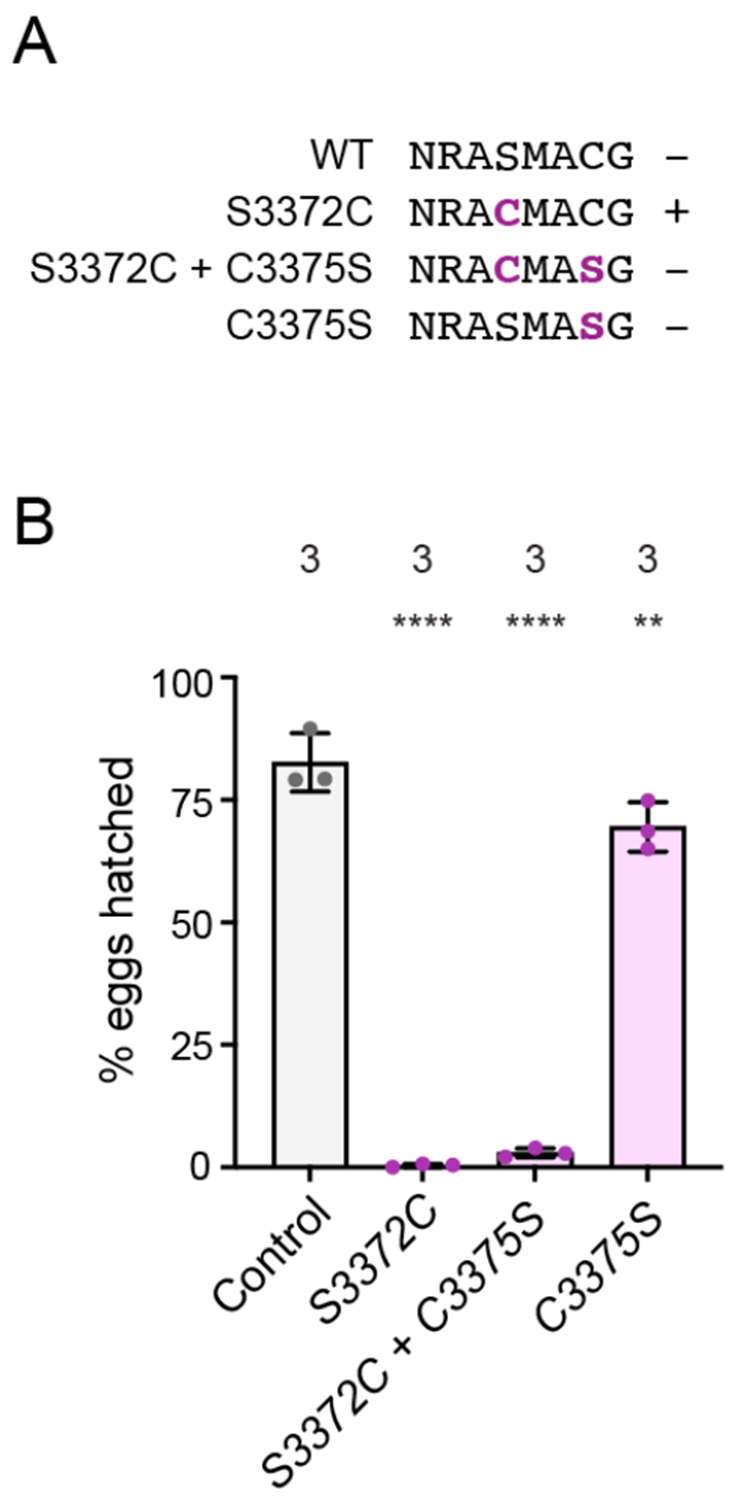
The S3372C embryonic arrest phenotype is not caused by ectopic disulfide bonding in Dhc. (A) Sequences of relevant regions of wild-type (WT), S3372C, S3372C + C3375S, and C3375S *Drosophila* Dhc. Mutated residues are shown in magenta. Potential for intramolecular disulfide bond formation is indicated with a plus. (B) Quantification of hatching rate of eggs laid by mated females of the indicated genotypes. Columns show mean values per egg collection; error bars represent S.D.; circles are values for individual egg collections. Number of collections per genotype (each from an independent cross; 245 – 736 eggs per collection) is shown above bars. Control genotype was homozygous for a wild-type *Dhc* allele recovered from the same CRISPR-Cas9 mutagenesis experiment that generated the *Dhc* mutant alleles. Evaluation of statistical significance (compared to control) was performed with a 1-way ANOVA with Dunnett’s multiple comparisons test: ****, P<0.0001; **, P<0.01.

The above structural analysis also revealed that, whilst there is no nearby cysteine on the α/β-tubulin dimer, there is a conserved cysteine at the base of CC2 in dynein-1 proteins (C3375 in *Drosophila*) that is in close proximity to the serine mutated in the *S3372C* strain (Figure 3B and C). These observations led us to hypothesize that the S3372C mutation results in an ectopic disulfide bond between this residue and C3375. There is a global increase in oxidation at the *Drosophila* egg-to-embryo transition (Petrova et al, 2018), which could conceivably trigger disulfide bonding at this site. Such an event could impair dynein’s dynamics specifically in the embryo and thereby explain arrest at this stage. To test this notion, we used CRISPR/Cas9-mediated HDR in *Drosophila* to change S3372 to cysteine and C3375 to serine in the same polypeptide, thus eliminating the potential for a disulfide bond at these positions (Figure 4A). Like *S3372C* females, females homozygous for this allele (*S3372C + C3375S*) were almost completely infertile (Figure 4B). This effect was not associated with the C3375S change, as females with this mutation had only modestly reduced fertility (Figure 4B). Thus, disulfide bonding with C3375 does not account for the embryonic arrest caused by S3372C.

### S3372C causes metaphase arrest of early nuclear divisions

**Figure 5.**
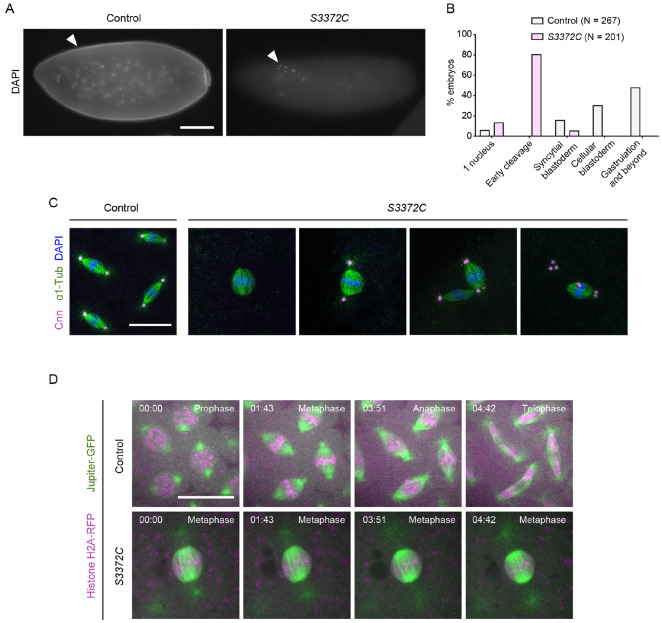
S3372C causes metaphase arrest in the embryo. (A) Example wide-field images of fixed embryos from a 2 to 4-h egg collection from mated control (*yw* strain) and *S3372C* mothers stained with DAPI (arrowheads show DNA staining). (B) Quantification of stages of control and *S3372C* embryos from 2 to 4-h egg collections. N is number of embryos scored. The ‘1 nucleus’ category includes potentially unfertilized eggs. (C) Example confocal images of mitotic spindles in fixed control and *S3372C* embryos (Z-projection). Cnn, Centrosomin (centrosome marker); α1-Tub, α1-Tubulin (microtubules). (D) Example stills from time series (single focal plane) of control and *S3372C* embryos acquired during preblastoderm cycles. Jupiter-GFP and His2Av-mRFP label microtubules and chromatin, respectively. Timestamps are min:s. Scale bars: A, 100 μm; C and D, 20 μm.

Our finding that S3372C does not destabilize Dhc protein in the embryo or cause ectopic disulfide bonding points to a more nuanced effect on dynein activity, possibly related to a specific function during development. To elucidate which motor function is affected by this mutation, we investigated the embryonic phenotype in more detail. DNA staining of fixed embryos revealed that the vast majority of embryos from *S3372C* homozygous mothers did not reach syncytial blastoderm stages, with ~80% arresting during early nuclear cleavage cycles (Figure 5A and B). Development of *S3372C* embryos to this stage was dependent on fertilization because only a single nucleus was observed in unfertilized mutant eggs (Figure S4A), as is the case in unfertilized wild-type eggs (Loppin et al, 2015). Arrest during early cleavage stages was also typical for embryos from *S3372C/-* mothers (Figure S4A), demonstrating the causal nature of the MTBD mutation. In contrast, embryos from *F579Y* mothers developed normally to the blastoderm stage (Figure S4A). The partially penetrant *F579Y* hatching defect observed previously (Figure 2A) must therefore be due to problems arising later in development.

To visualize the mitotic apparatus of *S3372C* mutant embryos, we additionally stained them for markers of centrosomes and microtubules. This revealed that the vast majority of mitotic spindles in S3372C embryos were arrested in metaphase (Figure 5C and Figure S4B). Moreover, we found that, whilst 70% of spindles in the mutants had centrosomes, only 10% had the canonical arrangement of one centrosome at each pole (Figure S4B). The other mutant spindles had a range of abnormalities in centrosome arrangement, including missing or supernumerary centrosomes at one pole, or detachment of centrosomes from the spindle (Figure 5C and S4B). Time-lapse analysis of mitosis in embryos that had fluorescently-labeled microtubules in combination with either fluorescently-labeled histones (Figure 5D and Movie S1) or centrosomes (Figure S4C and Movie S2) confirmed that S3372C causes metaphase arrest and defects in centrosome arrangement. The observation that some metaphase-arrested spindles had normal positioning and numbers of centrosomes (Figure S4B) suggested that altered centrosome arrangement is not primarily responsible for the metaphase arrest of *S3372C* spindles. Instead, a failure to progress to anaphase may uncouple mitotic cycles from centrosome duplication cycles, a scenario previously observed upon inhibition of several mitotic regulators (Archambault & Pinson, 2010; Defachelles et al, 2015; McCleland & O'Farrell, 2008).

The above analysis shows that the S3372C mutation blocks anaphase onset of mitotic spindles in the early embryo. To investigate if all dynein functions are affected at this stage of development, we examined the maintenance of the mitotic phase (M-phase) of polar bodies. Dynein has been implicated in this process through analysis of embryos in which the function of BicD or another dynein-associated protein, Rod, is disrupted (Defachelles et al, 2015; Vazquez-Pianzola et al, 2022). In a sizeable fraction of BicD- or Rod-deficient embryos, the polar body is not arrested in metaphase as judged by decondensation of the DNA, as well as the absence of phosphorylated histone H3 and the SAC component BubR1. We saw no such defects in *S3372C* embryos (Figure S5A and B), indicating that dynein’s function in maintaining M-phase of the polar body is not impaired. Thus, the S3372C mutation appears to only affect a subset of dynein functions in the early embryo that include – and are perhaps limited to – the progression of mitotic spindles from metaphase to anaphase.

### S3372C increases dynein accumulation in the vicinity of the kinetochore

**Figure 6.**
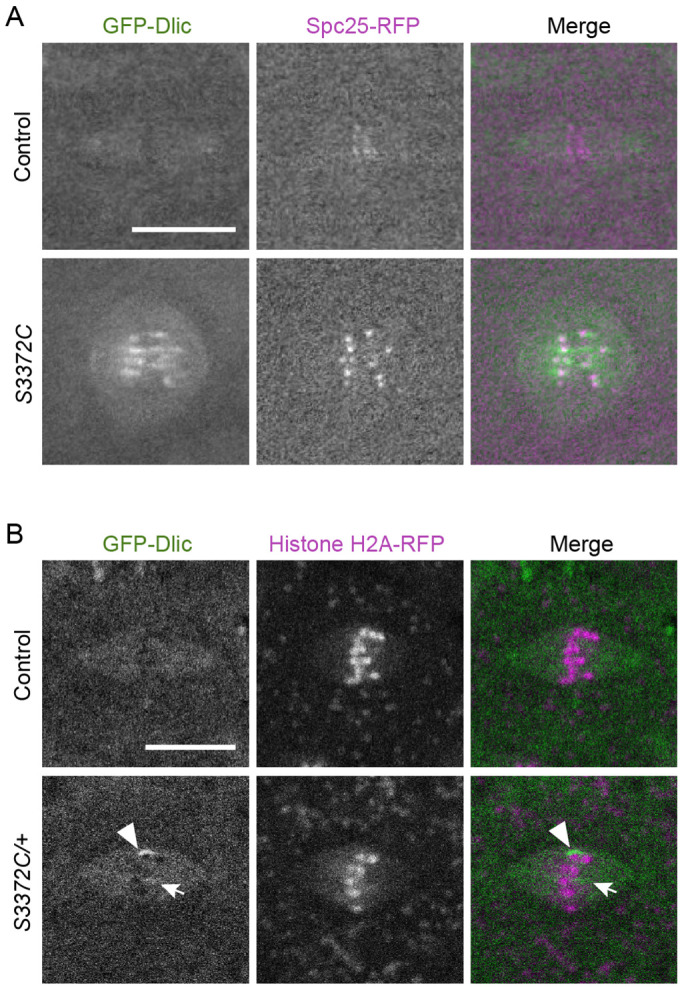
S3372C cause ectopic accumulation of dynein in the vicinity of kinetochores. (A, B) Example confocal images (Z-projections) of mitotic spindles in live control and (A) *S3372C* and (B) *S3372C/+* embryos with fluorescently-labeled dynein (GFP-Dlic). Embryos additionally contain markers of kinetochores (Spc25-RFP; A) and chromatin (Histone H2A-RFP; B). In B, arrow and arrowhead show examples of accumulation of dynein at the metaphase plate in *S3372C/+* embryos. Ectopic GFP-Dlic accumulation at ≥ 1 kinetochore was observed in 100% (21/21) of *S3372C* embryos, 43% (10/23) of *S3372C/+* embryos and 0% (20/20) of control embryos. Scale bars: 10 μm.

To attempt to narrow down how the S3372C mutation causes metaphase arrest of mitotic spindles, we investigated its effect on the distribution of the motor complex on the mitotic apparatus. This was achieved by comparing the localization of a GFP-tagged Dynein light intermediate chain subunit (GFP-Dlic) in embryos laid by wild-type and *S3372C* homozygous females. In wild-type embryos, GFP-Dlic was associated with the spindle for the duration of the mitotic cycle with a transient, weak enrichment at kinetochores during prometaphase and metaphase (Figure 6A and Movie S3). This pattern is in keeping with previous observations of dynactin localization during embryonic mitosis (Wojcik et al, 2001). In contrast, all *S3372C* mutant spindles had a very strong accumulation of GFP-Dlic signal in the vicinity of the kinetochores, which often extended along the neighbouring microtubules (Figure 6A and Movie S3). The mutant embryos did not, however, have an overt enrichment of GFP-Dlic on other regions of the spindle apparatus, including the spindle poles or centrosomes (Figure 6A and Movie S3). To test if the abnormal accumulation of dynein at the kinetochore region of mutant embryos is solely an indirect effect of the metaphase arrest, we examined GFP-Dlic localization in embryos of *S3372C/+* mothers, which have no apparent defects in mitosis. Unlike the wild type, a fraction of these embryos (43%) had ectopic kinetochore association of dynein (Figure 6B), albeit less strong than in embryos from *S3372C* homozygous mothers and only at a subset of kinetochores. Collectively, these observations suggest that the S3372C mutation can directly influence kinetochore-related functions of dynein.

### S3372C blocks anaphase progression in a SAC-independent manner

The above findings prompted us to examine kinetochore-associated roles of dynein in the mutant embryos in more detail. Functions of dynein at the kinetochore are closely linked with silencing of the SAC (Gassmann, 2023). This checkpoint delays metaphase-anaphase transition by inhibiting co-activators of the anaphase promoting complex/cyclosome (APC/C) ubiquitin ligase until chromosomes have been successfully biorientated (Musacchio, 2015).

**Figure 7.**
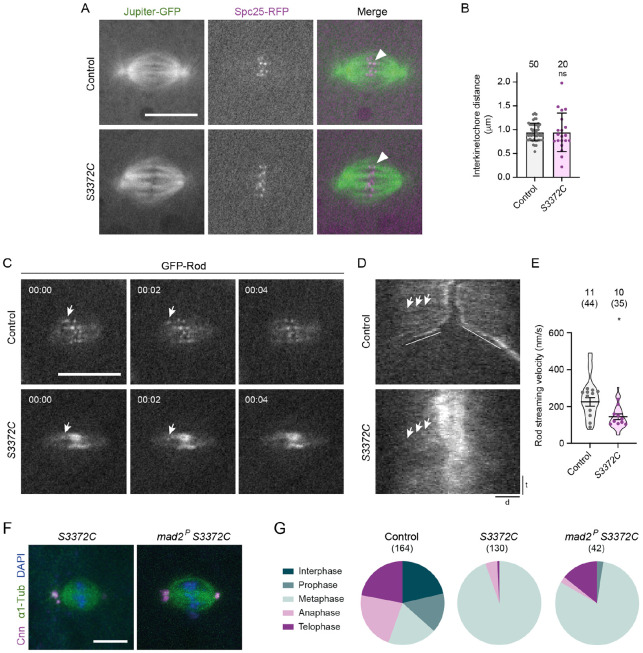
S3372C blocks mitosis in a SAC-independent manner. (A) Example confocal images of mitotic spindles (single focal plane) in live control and *S3372C* embryos that have fluorescently-labeled microtubules (Jupiter-GFP) and kinetochores (Spc25-RFP). Arrowheads, examples of association of bundled microtubules with a kinetochore. (B) Quantification of interkinetochore distance (using centroids of Spc25-RFP signals) in live control and *S3372C* metaphase spindles. Columns show mean values; error bars represent S.D.; circles are values for individual kinetochore pairs. Number of kinetochore pairs analyzed (from 7 control and 8 *S3372C* embryos) is shown above bars. (C) Example stills from a time series (single focal plane) showing streaming (arrows) of GFP-Rod particles in control and *S3372C* embryos. Timestamps are min:s. (D) Example kymographs showing GFP-Rod streaming (e.g. arrows). d, distance; t, time. Diagonal lines in control label movements of kinetochores in anaphase. (E) Quantification of GFP-Rod streaming velocity. Violin plots show values for individual motile Rod particles and circles show mean values per embryo. Horizontal lines shown mean ± S.D. of values for individual motile particles. Numbers without parentheses above bars are number of embryos, with total numbers of particles given in parentheses. (F) Example confocal images of metaphase spindles from fixed embryos of *S3372C* and *mad2*^*P*^, *S3372C* mothers stained as indicated (Z-projections). (G) Incidence of mitotic stages in control, *S3372C* and *mad2*^*P*^, *S3372C* embryos (25 – 130 minute collection). Numbers in parentheses indicate number of nuclei or spindles counted per genotype; data are from 34 control, 49 *S3372C* and 33 *mad2*^*P*^, *S3372C* embryos, with no more than 5 randomly selected nuclei or spindles analyzed per embryo. In B and E, statistical significance was evaluated with an unpaired t-test (in E, comparisons were between mean values per embryo). *, P<0.05; ns, not significant. Scale bars: A and C, 10 μm; D distance, 2 μm; D time, 30 s.

One function of dynein in this context is to generate end-on attachments of kinetochores with microtubule plus ends, which is a prerequisite for SAC silencing (Bader & Vaughan, 2010; Barisic & Maiato, 2015). In *S3372C* embryos, the ends of microtubule bundles were closely apposed with the kinetochore-associated proteins Spc25-RFP (Figure 7A) and the dynein activating adaptor Spindly (Figure S5C). This observation indicates that metaphase arrest in the mutants is not due to a failure of kinetochores to engage with microtubule plus ends. However, with the resolution available within the *Drosophila* embryo it was not possible to tell if the number or orientation of kinetochore-microtubule attachments were established correctly.

Dynein has also been implicated in creating tension between sister kinetochores in both mammals and flies (Siller et al, 2005; Varma et al, 2008; Yang et al, 2007). This process contributes to SAC inactivation in several systems, potentially by stabilizing kinetochore-microtubule attachments (Maresca & Salmon, 2010). To assess interkinetochore tension, we measured the distance between sister kinetochores using the centroids of Spc25-RFP signals. This analysis revealed that, whilst there was no difference in the mean interkinetochore distance between wild-type and *S3372C* metaphase spindles (Figure 7B), there was substantially more variability in this metric in the mutant condition (Levene test, P = 0.001). These data raise the possibility that the S3372C mutation results in variable levels of tension between kinetochore pairs.

A particularly well-characterized mitotic role of dynein in silencing the SAC is transporting checkpoint proteins away from kinetochores along the attached microtubules (so-called ‘streaming’; Basto et al, 2004; Gassmann et al, 2010; Griffis et al, 2007; Hinchcliffe & Vaughan, 2017; Howell et al, 2001; Wojcik et al, 2001). Dynein co-operates in this process with Spindly, which interacts with the Rod-Zwilch-Zeste White 10 (RZZ complex) to link SAC components to the motor (Gama et al, 2017; Griffis et al, 2007; Mosalaganti et al, 2017). To assess the transport of SAC components in *S3372C* embryos, we monitored the behavior of GFP-tagged Rod using time-lapse imaging. GFP-Rod showed an abnormal build-up at the kinetochore in *S3372C* mutants (Figure 7C and D), consistent with the previously observed strong accumulation of dynein at this site (Figure 6A). However, streaming of GFP-Rod could be observed in the mutant embryos, albeit at a significantly lower velocity than in the wild-type condition (Figure 7C - E and Movie S4).

It is conceivable that alterations in any one of kinetochore-microtubule attachments, tension between sister kinetochores, and dynamics of Rod streaming (or a combination of these scenarios) are sufficient to prevent SAC inactivation in *S3372C* embryos and thereby cause metaphase arrest. To determine if the SAC might remain active in the mutant embryos, we examined the distribution of Cyclin B, which is degraded by APC/C once the checkpoint is satisfied (reviewed by Huang & Raff, 1999; Murray, 1995; Musacchio, 2015). Whereas Cyclin B was barely detectable in the vicinity of the spindle apparatus in late metaphase and anaphase in wild-type embryos, it was associated with metaphase-arrested *S3372C* spindles (Figure S6A). Persistence of Cyclin B on the mutant spindles was not due to a failure to recruit Cdc20/fzy – the co-activator of APC in early embryos – to its sites of enrichment at the centrosome and kinetochore (Raff et al, 2002) (Figure S6B). These data are consistent with the S3372C mutation blocking the metaphase-anaphase transition by preventing SAC silencing.

To test if this is actually the case, we introduced a null mutation in the gene encoding the checkpoint protein Mad2 into the *S3372C* background. Because the SAC is inactive in *Mad2* mutants (Buffin et al, 2007; Defachelles et al, 2015), the *S3372C* mitotic arrest should be rescued in the double mutants if it is primarily caused by a failure to alleviate SAC inhibition. However, this was not the case – whilst there was an increase in the proportion of spindles in telophase in *Mad2 S3372C* double mutant embryos (14.3% vs 0.8% in *S3372C* single mutant embryos; Fisher’s exact test, P <0.001), the vast majority of spindles still arrested at metaphase (Figure 7F and G). Although this result does not rule out the MTBD mutant impairing SAC silencing through the mechanisms described above, it suggests that this can make, at best, a minor contribution to the mitotic arrest phenotype. Thus, our data point to a novel, SAC-independent function of dynein that is required for the metaphase to anaphase transition in embryos.

### The S3372C mutation specifically affects dynein behavior under load

Next, we attempted to elucidate how the S3372C mutation selectively affects anaphase progression. We first considered the possibility that the mutation impairs the interaction of dynein with a specific tubulin isotype that is important for this process. *Drosophila* has four α-tubulin isotypes and four β-tubulin isotypes (Nielsen et al, 2010). One of these proteins, α4-tubulin (also known as α-tubulin67C or maternal α-tubulin), is only expressed during the first two hours of embryogenesis (Matthews et al, 1989) and is known to co-operate with the ubiquitously expressed α1-tubulin (also known as α-tubulin84B) to facilitate early nuclear divisions (Mathe et al, 1998; Matthews et al, 1993; Venkei et al, 2006). These observations make α4-tubulin the most likely tubulin isotype to mediate the specific effects of the S3372C mutation. Immunostaining revealed that whereas α1-tubulin was present throughout the spindle apparatus, α4-tubulin was enriched at the spindle poles (Figure S7A). Thus, α4-tubulin does not seem well placed to mediate the effects of S3372C dynein at the kinetochore. More significantly, although α4-tubulin and α1-tubulin have only 67% amino acid identity, the dynein-binding region of the two proteins is identical (Figure S7B). Thus, it seems unlikely that the S3372C mutation specifically affects the interaction of dynein with α4-tubulin.

These observations led us to explore the alternative idea that S3372C alters a general aspect of dynein behavior that is particularly important for anaphase progression in embryos. We therefore set out to determine the effects of the S-to-C mutation on the activity of purified dynein complexes *in vitro*. To this end, the equivalent mutation (S3386C) was introduced into the full human dynein complex, for which a recombinant expression system is available (Schlager et al, 2014; Figure S8A). As expected from its position in the MTBD, this mutation did not prevent the association of the heavy chain with the other dynein subunits (Figure S8B).

**Figure 8.**
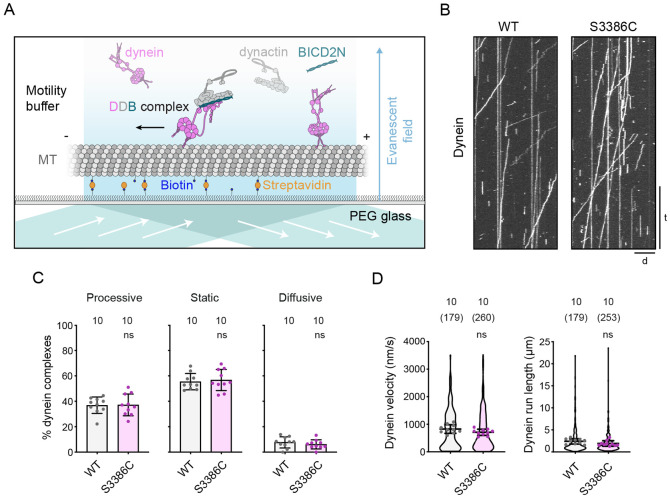
The S-to-C mutation does not alter motility of an isolated dynein-dynactin-activating adaptor complex. (A) Diagram of TIRF microscopy-based *in vitro* motility assay using an assembly of TMR-labeled dynein, dynactin and BICD2N. In the absence of dynactin and BICD2N, dynein is autoinhibited and not capable of long-range transport. MT, microtubule; PEG, polyethylene glycol (used for passivation). (B) Example kymographs of wild-type (WT) or S3886C TMR-ådynein motility in the presence of dynactin and BICD2N in the assembly mix. There were no consistent differences in the intensity of dynein signals between the wild type and mutant across the experiments. Microtubule minus end is to the left. d, distance; t, time. Scale bar: distance, 5 μm; time, 20 s. (C, D) Quantification of (C) percentage of microtubule-associated dynein complexes that exhibit processive, static or diffusive behavior and (D) velocity and run length of the processive dynein fraction. In C, columns display mean values for individual microtubules; error bars are S.D.; circles are values for individual microtubules. Numbers above each column indicate the total number of microtubules in each case. In D, violin plots show values for individual motile DDB complexes and circles show mean values per microtubule. Horizontal lines shown mean ± S.D. of values for individual DDB complexes. Numbers without parentheses above bars are number of microtubules, with total numbers of motile DDB complexes given in parentheses. In C and D, statistical significance was evaluated with an unpaired t-test (in D, comparisons were between mean values per microtubule). ns, not significant. Data were summed from 2 experiments per condition. See Figure S8C and D for histograms of velocity and run length.

We first monitored the movement of tetramethylrhodamine (TMR)-labeled wild-type and S3386C mutant dynein complexes along immobilized pig brain-derived microtubules in the presence of dynactin and BICD2N using total internal reflection fluorescence (TIRF) microscopy (Figure 8A). It is in this assay that the human disease-associated mutations were found to significantly impair dynein activity (Hoang et al, 2017). In contrast, the S-to-C mutation had no effect on the frequency of processive movements of dynein, or the distance or velocity of these events (Figure 8B - D and Figure S8C and D). Thus, the novel MTBD mutation does not perturb the translocation of isolated dynein-dynactin-activating adaptor complexes along microtubules.

**Figure 9.**
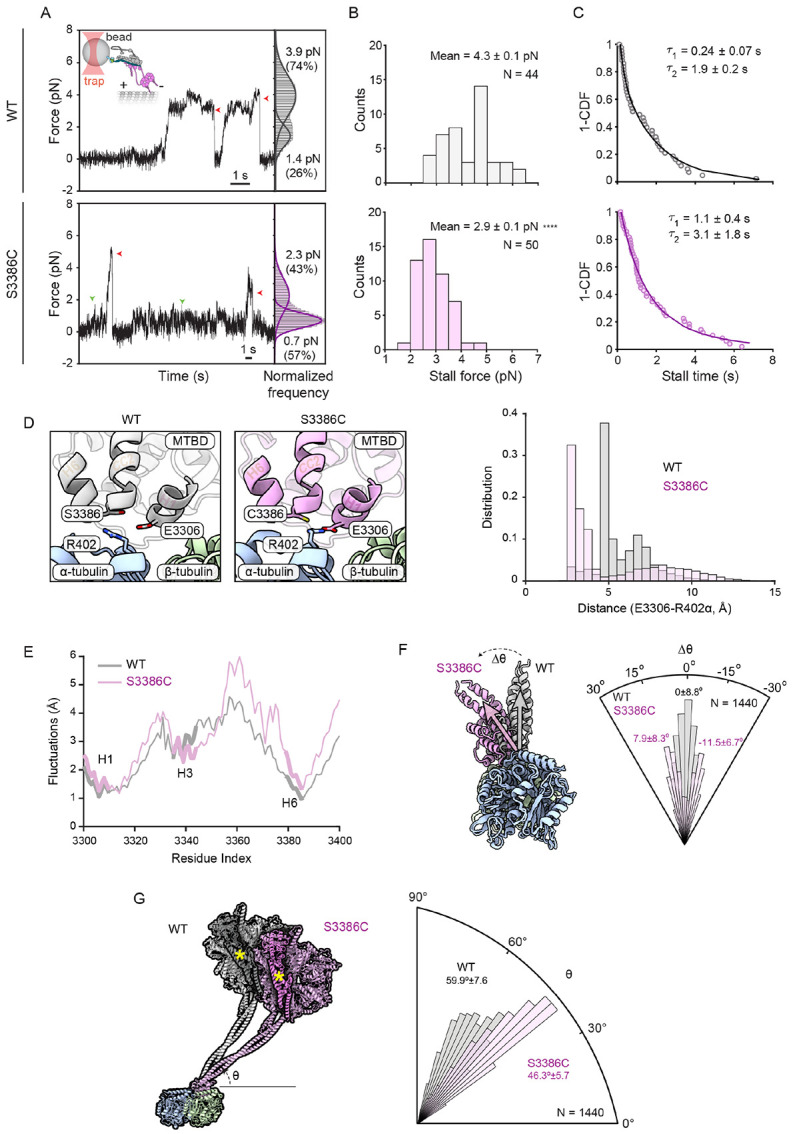
The S-to-C mutation alters dynein-dynactin-activating adaptor behavior under load and the position of the dynein MTBD and stalk relative to the microtubule. (A) Left, representative traces of beads driven by a single wild-type (WT) or S3386 DDB complex in a fixed optical trapping assay. Traces were downsampled to 250 Hz. Red arrowheads show detachment of the motor from a microtubule after the stall; green arrowheads show pausing of the mutant dynein at low forces. Inset cartoon shows how DDB is attached to beads from its BICD2N subunit via GFP-antibody linkage. Right, normalized histograms showing the probability of a dynein-driven bead sampling different forces under the trap. Dwelling of the beads at 0 pN force when they are not tethered to the microtubule was subtracted from the histogram. Solid curves represent a multiple Gaussian fit to calculate the mean and percent population of the 2 peaks. (B) Stall forces of DDB complexes with WT and mutant dynein (errors are S.E.M.; N is number of stalls). Statistical significance was evaluated with an unpaired t-test. ****, P<0.0001. (C) Inverse cumulative distribution (1-CDF) of motor stall times. Time constants (τ ± S.E.M.) were calculated from a fit to a double exponential decay (solid curves). In A – C, data are from 11 beads in 5 independent experiments for WT and from 14 beads in 7 independent experiments for mutant. (D) Left, example snapshots of E3306(dynein) and R402(α-tubulin) positioning in MD simulations of the WT and S3386C human dynein MTBD plus a portion of the stalk in the presence of the α/β-tubulin dimer. Right, distance distributions of E3306(dynein)-R402(α-tubulin) in WT and S3386C dynein simulations. (E) Fluctuations of WT and S3386C MTBD and portion of the stalk with respect to the microtubule in MD simulations. H1, H3, and H6 helices are highlighted with thicker lines. (F) Left, superimposition of examples from simulations with the WT and S3386C MTBD plus a portion of stalk when viewed down the longitudinal axis of the microtubule. Gray and magenta vectors show most frequent MTBD orientation for WT and S3386C, respectively. Right, angular orientation histogram (normalized frequency) for the MTBD around the longitudinal axis of the microtubule in simulations. The WT distribution exhibited a single peak (assigned a value of 0), whereas the S3386C mutant exhibited a double-peaked distribution (peaks of fitted curves ± S.D. are shown). (G) Left, superimposition of examples from simulations with WT and S3386C MTBD plus a portion of stalk when viewed facing the longitudinal axis of the microtubule. The remainder of the dynein motor domain structure (PDB, 7Z8F; Chaaban & Carter, 2022) was superimposed on the simulations to show predicted position of the linker (darker shading and yellow asterisk) and AAA+ ring. Right, angular orientation histogram (normalized frequency) for the stalk relative to the longitudinal axis of the microtubule in simulations (peaks of distributions ± S.D. are shown). In F and G, N is number of trajectories for each type of complex.

The absence of cargo in the above assays meant that dynein was operating under minimal load. To determine if the S-to-C mutation influences dynein performance under resistive loads, we quantified force production by the motor using high-resolution optical trapping (Figure 9A). Polystyrene beads were sparsely decorated with BICD2N-GFP via a GFP antibody, incubated with dynactin and either wild-type or S3386C dynein, and brought near a surface-immobilized microtubule using an optical trap (Belyy et al, 2016). Fixing the position of the trap while the motor carries the bead along the microtubule allows quantification of the force at which the bead detaches from the microtubule after a brief stall (referred to as the stall force). Whilst performing these experiments we noticed that, whereas the wild-type DDB complex moved rapidly along the microtubule at low resistive forces (~1 pN), the S3386C version had a tendency to pause in these conditions (Figure 9A; e.g. green arrowheads). Following these pauses, the mutant DDB either detached from the microtubule or continued to move forward. These data indicated that S3386C dynein is sensitive to external load. Confirming this notion, whereas the stall force of beads driven by wild-type DDB was 4.3 ± 0.1 pN (mean ± S.E.M.), consistent with previous observations (Belyy et al, 2016), the value was reduced to 2.9 ± 0.1 pN for the mutant (Figure 9B). Thus, the S-to-C mutation reduces dynein’s peak force production by one-third. The mutant complexes also had longer stall times than the wild type (Figure 9C), which may be related to the stalling of the motor at lower resistive forces. These results show that mutant dynein exhibits defective motility in the presence of external load.

### S3372C modulates dynein’s microtubule interaction and stalk positioning

Remarkably, the effects of the S-to-C change on dynein’s function in embryonic mitosis and its behavior under load stem from the substitution of a single oxygen atom for a sulfur atom within the ~90,000-atom Dhc polypeptide. To evaluate the effects of the S-to-C change on the structure and dynamics of the MTBD, as well as its interaction with the microtubule, we performed all-atom MD simulations of the human wild-type and S3386C MTBD together with a portion of the stalk bound to the α/β-tubulin dimer (modeled on the cryo-EM structure of Lacey et al, 2019). Four sets of 900-ns-long simulations were performed for each variant in the presence of explicit solvent.

Analysis of the ensemble of conformations in the simulation trajectories of the wild type revealed that S3386 did not make contact with the α/β-tubulin dimer. The residue did, however, frequently form hydrogen bonds with the main chain of residues in H6 of the MTBD (V3382) and the base of CC2 of the stalk (C3389) (Figure S8E and Table S1). Thus, the serine appears to stabilise the interaction of H6 with the stalk. In the simulations with the mutant dynein, the overall fold of the MTBD was not overtly different from the wild type. C3386 also did not make contact with tubulin and had a very similar frequency of interactions with V3382 and C3389 to that observed with S3386 (Figure S8E and Table S2). However, the mutation led to hydrophobic interactions with several neighboring residues in the MTBD that were not evident in the wild type (Figure S8Eand Table S3). This was accompanied by frequent formation of a salt bridge between E3306 within H1 of the MTBD and R402 of α-tubulin, which was seldom seen in the wild-type simulations (Figure 9D; frequency of 62.4% and 8.4% in mutant and wild-type simulations, respectively). The mutation also increased the magnitude of fluctuations of several elements of the MTBD relative to the microtubule (mean of 16.9% compared to the wild type), with those located between the end of H3 and the start of CC2 of the stalk showing the greatest effect (mean of 25.7% compared to the wild type) (Figure 9E). Thus, the S-to-C mutation alters the MTBD’s interaction with the microtubule, including by increasing the relative flexibility of the MTBD.

Further analysis demonstrated that, whilst the registry of the stalk was not sensitive to the S-to-C change, the mutation induced either clockwise or anticlockwise tilting of the MTBD and base of the stalk around the longitudinal axis of the α/β-tubulin dimer (Figure 9F; peak of angle distribution for the mutant 11.5° clockwise and 7.9° anticlockwise compared to the wild type). In addition, the angle of the stalk relative to the longitudinal axis of the tubulin dimer was decreased by the mutation (Figure 9G; peak of angle distribution 59.9° and 46.3° for wild-type and S3386C dynein, respectively). As discussed below, these changes in stalk positioning relative to the microtubule could account for the force-sensitive nature of the mutant motor complex.

## DISCUSSION

### Mutational analysis of Dhc *in vivo*

The diverse cellular roles of dynein can complicate functional analysis of the motor significantly. We set out to address this problem by combining mutagenesis in *Drosophila* with *in vitro* analysis of motor activity. We first assessed the phenotypes of disease-associated missense mutations in Dhc for which *in vitro* effects have already been defined. Abnormalities were only manifest in homozygous mutant *Drosophila*, whereas the equivalent mutations in human or mouse cause neurological disease when heterozygous (Poirier et al, 2013; Schiavo et al, 2013; Vissers et al, 2010; Willemsen et al, 2012). This may reflect the relatively short length of *Drosophila* neurons making them less sensitive to partially impaired cargo transport. Nonetheless, the homozygous *Drosophila* phenotypes provide valuable information about the consequences of *Dhc* disease-associated mutations in an animal model, and thereby extend previous work on these lesions *in vitro* (Hoang et al, 2017) and in budding yeast (Marzo et al, 2019). For example, our observation that disease-linked mutations can cause abnormal accumulation of synaptic vesicles and impaired retrograde transport of mitochondria in axons supports our hypothesis that they interfere with the motility of cargo-motor complexes in neurons (Hoang et al, 2017).

The scope of our study was increased by the recovery of novel *Dhc* alleles whilst generating the disease-associated mutations. Collectively, the phenotypic characterization of the disease-associated and novel mutations establishes an allelic series that will facilitate studies of dynein’s involvement in multiple processes during development and in adulthood. Some of the mutations we investigated cause phenotypes – synaptic vesicle accumulations, impaired mitochondrial transport, and short bristles – that were previously seen with classical hypomorphic mutations in *Dhc* or when the function of the gene is knocked down with RNAi (Gepner et al, 1996; Martin et al, 1999; Melkov et al, 2016; Vagnoni et al, 2016). Thus, these mutations may impair core dynein functions to varying degrees, rather than affect discrete processes. Our earlier finding that the disease-associated mutations reduce the motility of purified dynein-dynactin-activating adaptor complexes to different extents (Hoang et al, 2017) is consistent with this scenario.

In sharp contrast, the novel MTBD mutation, S3372C, only affects a subset of dynein functions. This allele does not cause visible abnormalities in adults, yet homozygous mothers are infertile because mitotic spindles in their embryos arrest at metaphase. Whilst some classical hypomorphic dynein alleles, as well as the F597Y mutation, also have maternal effects on embryogenesis, these only become evident at later embryonic stages and are accompanied by morphological phenotypes in the parents (Gepner et al, 1996; Melkov et al, 2016; Robinson et al, 1999; Wilkie & Davis, 2001; this study). Consistent with the normal appearance of *S3372C* adults, we found that the mutation does not impair dynein-dependent functions during mitosis in larval neuroblasts, or mitochondrial transport in axons of adult neurons. Collectively, these analyses demonstrate that S3372C disrupts a specific, maternal activity of dynein that is critical for early embryogenesis. The effect of S3372C contrasts sharply with that of the neighbouring R3370Q mutation, which causes zygotic mutants to arrest during larval stages. These findings validate the use of mutagenesis in flies to dissect the *in vivo* functions of the motor complex.

### The mechanistic basis of S3372C’s selective phenotype and force sensitivity

The observations described above raised the question of how the S3372C mutation has such a selective effect on Dhc activity. The finding that embryos of heterozygous *S3372C* mothers have no developmental defects is consistent with the allele causing a loss, rather than a gain, of dynein function. We have shown that neither stage-specific instability of Dhc protein nor ectopic disulfide bonding within it is responsible for the embryo-specific phenotype. We have also provided evidence that an altered interaction of dynein with the maternal α4-tubulin isotype is not a contributing factor. Whilst we cannot rule out the S-to-C change blocking a phosphorylation event on S3372 that is important for anaphase onset, this seems unlikely because the frequent hydrogen bonding of this sidechain with neighboring residues in H6 and CC2 means it should not be readily accessible to a kinase. Indeed, there is no evidence that S3372 is post-translationally modified in previous proteome-wide studies of embryos from multiple *Drosophila* species (Hu et al, 2019) or our ongoing analysis of dynein complexes immunoprecipitated from *D. melanogaster* embryos.

However, using high-resolution optical trapping, we observed a striking effect of the S-to-C mutation on the behavior of dynein-dynactin-activating adaptor complexes operating with a resistive load. Whereas motility was normal in the absence of load, mutant complexes exhibited excessive pausing when exposed to a resistive force, as well as a substantially reduced peak stall force. Therefore, the most parsimonious explanation for the restricted *in vivo* phenotype is that the metaphase to anaphase transition in the embryo needs dynein to work in a specific load regime that is problematic for the mutant motor complex. Consistent with this notion, we observed a reduced velocity of dynein-driven motion of GFP-Rod away from the metaphase plate in *S3372C* mutant embryos.

Our MD simulations of the human Dynein heavy chain’s interaction with the microtubule allow us to speculate how the mutation causes force sensitivity. Our data indicate that replacement of serine’s hydroxyl group with the more hydrophobic sulfhydryl group of cysteine (Catalano et al, 2021) causes several new hydrophobic interactions with neighbouring residues, which are accompanied by increased flexibility of the MTBD, as well as repositioning of the stalk, relative to the microtubule. Our analyses suggest that the MTBD and stalk in the mutant dynein tilt abnormally in both the anticlockwise and clockwise direction in the plane perpendicular to the longitudinal axis of the microtubule. Thus, the stalk appears to be more mobile in this plane in the mutant, which may reduce dynein’s ability to overcome an opposing force. The mutation also reduced the angle between the stalk and the longitudinal axis of the microtubule, a change that is expected to increase the distance the linker moves with respect to this axis during its powerstroke. This would mean that more work needs to be done against the opposing horizontal force during the linker swing, which could contribute to the reduced force output. An interesting implication of these ideas for wild-type dynein mechanism is that the MTBD plays an important role in maximising motor performance under load by controlling stalk orientation.

### Insights into dynein function in mitosis

As described in the Introduction, a prime example of the difficulties of disentangling dynein’s *in vivo* functions is in mitosis, where the motor has been implicated in a wide range of processes. Partial knockdowns with RNAi, function-blocking antibodies and small molecule antagonists have each been used to circumvent the cell lethality that results from complete loss of dynein activity. Unfortunately, each of these approaches simultaneously targets many aspects of motor function, which can indirectly affect the specific mitotic process being studied. In the *Drosophila* embryo, dynein’s roles in mitosis have also been investigated with hypomorphic mutations (Robinson et al, 1999). However, in addition to being likely to affect multiple dynein-dependent events, these mutations have weak mitotic phenotypes since sufficient protein function must remain to produce viable mothers. Our discovery of a missense mutation that strongly affects nuclear divisions in the embryo without disrupting other dynein functions offers a unique tool to study the mitotic roles of the motor.

Although we cannot exclude an indirect effect of the S3372C mutation on dynein’s functions in other parts of the spindle apparatus, such as the poles and centrosomes, the build-up of dynein at the kinetochore, as well as slower transport of Rod away from this site, suggest that impaired kinetochore functions of dynein make an important contribution to the metaphase arrest. In light of our discovery of force sensitivity imparted by the S-to-C mutation, we can speculate that tight bundling of microtubules at kinetochores in embryonic spindles, or other physical constraints of this environment, provide a strong opposition to motility that cannot be overcome by the mutant motor. In such a scenario, the ectopic build-up of S3372C dynein in the vicinity of the kinetochore and associated microtubules may be the manifestation of the increased pausing of the motor complex observed *in vitro* under resistive loads. According to this view, the failure of S3372C to block mitosis in other cell types, include L3 neuroblasts, could reflect differences in the forces encountered by dynein near the kinetochore. Alternatively, there may be redundant mechanisms for initiating anaphase in these systems.

Remarkably, the MTBD mutation does not appear to block anaphase progression in embryos by preventing the well-characterized role of kinetochore-associated dynein in silencing the SAC, as the defect persists when the checkpoint is inactivated by mutation of Mad2. Collectively, these observations indicate that kinetochore dynein has a novel role in licensing the transition from metaphase to anaphase. We found that this function is not associated with a failure to localize the APC/C co-activator Cdc20/fzy to the spindle apparatus. Dynein may therefore directly promote coupling of Cdc20/fzy to APC/C. Alternatively, APC/C activation may be triggered indirectly by another kinetochore-associated process that depends on the motor. For example, the apparent variability in tension between sister kinetochores in *S3372C* embryos, which could reflect abnormal force generation by the mutant motor complex, might prevent APC/C activation through the complex series of signalling events that respond to chromosome biorientation (Fujimitsu & Yamano, 2021; Krenn & Musacchio, 2015; Liu et al, 2012; McVey et al, 2021). It is also possible that force production by dynein plays a physical role in separating sister chromosomes downstream of APC/C activation, or that the ectopic accumulation of dynein and associated proteins at the kinetochore in the mutant embryos impairs engagement of other important factors with this structure. Investigating these, as well as other, potential explanations for the metaphase block in the *Drosophila* embryo will be the goal of future studies.

### Outlook

As well as pointing to novel mechanisms controlling anaphase progression, the S-to-C mutation in the MTBD may be valuable in other contexts. As the mutated serine is widely conserved, including in dynein-2 and axonemal dyneins, its substitution with cysteine may allow load-dependent functions of dynein family members to be dissected in other contexts. Moreover, if we are correct and the mutation causes force-responsive dwelling of the motor on microtubules *in vivo*, the location of the mutant dynein complexes may act as a reporter of subcellular regions and events where the motors are experiencing high load. This information could be useful for producing quantitative models of motor behavior *in vivo*. We also anticipate that our results will stimulate further efforts to dissect the function of cytoskeletal motors by genetic manipulation of specific mechanical properties. Whilst *Drosophila* is an attractive organism in which to pursue this work because of the ease of gene editing and ability to study motor function in cells within tissues, the same approach can of course be taken in other systems.

## MATERIALS AND METHODS

### *Drosophila* culture and existing strains

*Drosophila* strains were cultured on Iberian fly food (5.5% [w/v] glucose, 5% [w/v] baker’s yeast, 3.5% [w/v] organic wheat flour, 0.75% [w/v] agar, 0.004% [v/v] propionic acid, 16.4 mM methyl-4-hydroxybenzoate [Nipagin]) at 25°C and 50% ± 5% relative humidity with a 12h-light/12h-dark cycle. The following previously established strains and alleles were used in this study: *w*^*1118*^ (Bullock lab stocks); *yw* (Bullock lab stocks); *Dhc*^*null*^ (Fumagalli et al, 2021); *appl-* Gal4 (Torroja et al, 1999); *UAS-mito::GFP* (Pilling et al, 2006); *insc-Gal4>UAS-ChRFP::α-Tubulin* (Gallaud et al, 2022; Hartenstein et al, 2015); *His2Av::mRFP* (Heeger et al, 2005); *Jupiter::GFP* (Morin et al, 2001); *Asl::mCherry* (Conduit et al, 2015); *Spc25::mRFP* (Schittenhelm et al, 2007); *GFP::Dlic* (Pandey et al, 2007); *Dhc::3HA* (*Dhc* genomic rescue construct; Iyadurai et al, 1999); *GFP::Rod* (Basto et al, 2004); *GFP::fzy* (Raff et al, 2002); and *mad2*^*P*^ (Buffin et al, 2007). The *mad2*^*P*^ *S3372C* strain was generated by recombination. The presence of the *mad2* null allele in the *mad2*^*P*^ and *mad2*^*P*^ *S3372C* strains was confirmed by PCR and Sanger sequencing.

### CRISPR/Cas9-mediated knock-in of *Dhc* mutations

Mutations in the *Drosophila Dhc64C* gene were generated using previously established procedures (Port & Bullock, 2016; Port et al, 2014). Briefly, pCFD3 plasmids were generated that express, under the control of the U6:3 promoter, single gRNAs that target *Dhc* close to the codon to be mutated (see Table S4 for sequences of oligonucleotides used for gRNA cloning). For the experiments designed to produce F579Y, R1951C, R3370Q, S3372C + C3375S, C3375S and H3808P, transgenic strains expressing the gRNAs were established, with males of these strains crossed to *nos-cas9* females (CFD2 strain; Port et al, 2014) to produce *nos-cas9/+; gRNA/+* embryos. These embryos were injected with a 500 ng μl^−1^ solution of a donor oligo (Ultramer, IDT) that codes for the desired missense mutation (see Table S4 for donor sequences). In cases where the mutation would not disrupt targeting by the gRNA, synonymous changes that prevent recutting of the modified allele by the Cas9/gRNA complex were also introduced (Table S4). For the generation of the other mutations, the above procedures were replicated, except the gRNAs were introduced by co-injection of the pCFD3-gRNA plasmid (100 ng μl^−1^ solution) with the donor oligo into *nos-cas9* embryos.

Flies containing the desired mutation were identified by sequencing of PCR products containing the target region, as described (Port & Bullock, 2016), with stocks established using balancer chromosomes. Other in-frame mutations, which resulted from imprecise repair of Cas9-mediated DNA cleavage, were also retained for phenotypic analysis. With the exception of *S3372C* and *R3370Q*, all mutations were isogenized by backcrossing to the *w*^*1118*^ strain for 6 – 10 generations. In addition, we balanced a chromosome in which *Dhc* had not been mutated during the CRISPR process. This ‘CRISPR WT’ strain was used as the control genotype for a subset of experiments.

### Assaying lethality and fertility

To assess lethality, flies heterozygous for the *Dhc* missense mutations (or the CRISPR WT chromosome) and balanced with TM6B were crossed together or with *Dhc*^*null*^*/TM6B* flies. Absence of non-TM6B adult offspring indicated developmental arrest of homozygotes or *trans*-heterozygotes. To assess the stage of development arrest, crosses were performed with stocks balanced with the fluorescent balancer *TM3 [actin5C>GFP]*. In these experiments, cohorts of homozygous (GFP-negative) embryos were transferred to plates containing apple-juice agar (1.66% [w/v] sucrose, 3.33% [w/v] agar, 33.33% [v/v] apple juice and 10.8 mM methyl-4-hydroxybenzoate), and the number of animals that survived until early L2, late L2, L3 and pupal stages scored through regular inspections. Genotypes that did not arrest before pupal stages but did not reach adulthood were classed as pupal lethal and this was confirmed in independent crosses with the TM6B balancer, which has the *Tb* marker that is visible at pupal stages.

Fertility of *Dhc* mutant and control females was assessed in crosses to wild-type (*w*^*1118*^) males 5 days after eclosion of the females. After 24 h, crosses were transferred to egg-laying cages capped with apple-juice agar plates and the proportion of total embryos that had hatched 28 – 48 h after egg-laying was recorded in multiple technical replicates over the next 3 days.

### Immunostaining

L3 wandering larvae were dissected and fixed in 4% formaldehyde as described (Hurd & Saxton, 1996). Embryos were collected, dechorionated, fixed at the interface of 4% formaldehyde and n-heptane, and devitellinized using standard procedures (Port et al, 2014). Larval preparations were washed in PBS/0.1% Tween (PBT) and blocked in 20% Western Blocking Buffer (Sigma Aldrich) in PBT, whereas embryos were washed in PBS/0.1% Triton X-100 (PBST) and blocked in 20% Western Blocking Buffer in PBST. Details of primary and secondary antibodies, including working dilutions, are provided in Table S5 and S6. Samples were mounted in Vectashield containing DAPI (Vector Laboratories). Segmental nerves were imaged with a Zeiss 780 laser-scanning confocal microscope using a 40x/1.3 NA oil-immersion objective. Embryos were typically imaged with a Zeiss 710 or 780 laser-scanning confocal microscope using a 63x/1.4 NA oil-immersion objective, the exception being the use of a Nikon T2 wide-field microscope equipped with a 20x/0.75 NA air objective for the initial documentation of the stage of embryonic arrest of *S3372C* mutants.

### Assessing mitochondrial transport in the wing nerve

Fly wing mounting, imaging and analysis were performed as described previously (Vagnoni & Bullock, 2016; Vagnoni et al, 2016). Briefly, CO2-anethetized male or female flies that had eclosed 2 days earlier were mounted on double-sided sticky tape and wings coated in Voltalef 10S halocarbon oil (VWR). Movements of mitochondria (labeled with mito-GFP under the control of the neuronal driver *appl-GAL4*) in the arch region of the wing nerve were visualized with an UltraVIEW ERS spinning disk system (PerkinElmer) equipped with an Orca ER Charge-coupled device (CCD) camera (Hamamatsu) using a 60x/1.2 NA oil-immersion objective on an IX71 microscope (Olympus). A single focal plane was imaged with an acquisition rate of 0.5 frames s^−1^ for 3 min. Images were processed and analyzed with ImageJ (Schneider et al, 2012). The genotypes of the image series were hidden from the experimenter using the BlindAnalysis macro (Steve Royle, University of Warwick), followed by image straightening, stabilization and manual tracking of motile mitochondria in MTrackJ, as described previously (Vagnoni & Bullock, 2016; Vagnoni et al, 2016). We additionally quantified the total number of mitochondria per 50 μm of the wing nerve and the proportion of these that underwent transport (i.e. had at least one continuous bout of net motion of ≥ 2 μm [a ‘run’]).

### Analysis of *Drosophila* neuroblast divisions

Brains of L3 larvae (~120 h after egg laying) were dissected in Schneider’s media (Sigma Aldrich) containing 10% foetal calf serum (ThermoFisher Scientific) and transferred to 50 μl wells of Ibidi Angiogenesis μ-Slides for live imaging. Mutant and control brains were imaged in parallel at 25°C. Z-series with a height of 20 μm and 1-μm spacing were acquired every 30 s using a spinning disk system consisting of a Leica DMi8 microscope equipped with a 63x/1.4 NA oil-immersion objective, a CSU-X1 spinning disk unit (Yokogawa) and an Evolve Electron-Multiplying CCD (EMCCD) camera (Photometrics). The microscope was controlled by Inscoper Imaging Suite software (Inscoper). Images were processed with Fiji (Schindelin et al, 2012).

### SDS-PAGE and immunoblotting

*Drosophila* embryo extracts were generated for immunoblotting as described (McClintock et al, 2018). SDS-PAGE and protein transfer to polyvinylidene difluoride (PVDF) membrane (Immobilon-P, Merck Millipore) was performed with the NuPAGE Novex and XCell II Blot Module systems (ThermoFisher Scientific) according to the manufacturer’s instructions. Membranes were blocked with 5% dried skimmed milk powder (Marvel) and washed in PBS/0.05% Tween-20. Details of primary and secondary antibodies, including working dilutions, are provided in Table S5 and S6. Secondary antibodies were detected with ECL Prime reagents (Cytiva Amersham) as instructed by the manufacturer.

### Sequence and structure analysis

Alignments of Dynein heavy chain and α-tubulin sequences were produced with ESPript 3.0 (Robert & Gouet, 2014; https://espript.ibcp.fr/ESPript/ESPript/). Visualization and analysis of experimentally determined MTBD structures was performed with PyMOL (version 2.5.1; Schrödinger) and ChimeraX 1.2.5 (Goddard et al, 2018; https://www.cgl.ucsf.edu/chimerax/). The cryo-EM structure of the mouse dynein-1 MTBD and portion of the stalk bound to the microtubule (PDB 6RZB; Lacey et al, 2019) was generated from a ‘cysteine-light’ dynein in which C3389 was mutated to alanine. The alanine residue was therefore substituted for the native cysteine in Figure 3B.

Structure predictions were performed using a local installation of ColabFold 1.2.0 (Mirdita et al, 2022), running MMseqs2 (Mirdita et al, 2019) for homology searches and Alphafold2 (Jumper et al, 2021) for predictions with 3 recycles. For the MTBD and stalk regions of different dyneins, predictions were performed with the following amino acid sequences: *D. melanogaster* Dhc (Uniprot ID P37276) 3212-3451, *H. sapiens* DYNC1H1 (Uniprot ID Q14204) 3227-3465, *H. sapiens* DYNC1H2 (Uniprot ID Q8NCM8) 2922-3162, and *H. sapiens* DYH7 (Uniprot ID Q8WXX0) 2614-2866. The top-ranking models were visualized in ChimeraX 1.2.5., with secondary structures colored based on their structural homology to mouse dynein (PDB 6RZB; Lacey et al, 2019).

### Live imaging of mitosis in *Drosophila* embryos

Dechorionated transgenic embryos with fluorescently-marked centrosomes, histones, microtubules, kinetochores, Dlic, Rod, or Fzy/Cdc20 were filmed under Voltalef 10S halocarbon oil (VWR) using the Ultraview ERS spinning disk system described above with a 60x/1.2 NA water-immersion objective or a Zeiss 710 laser scanning confocal with a 63x/1.4 NA oil-immersion objective. A single focal plane was imaged with an acquisition rate of 0.2 frames s^−1^ for up to 15 min to capture complete nuclear division cycles. To capture Rod streaming, an acquisition rate of 0.5 frames s^−1^ was used. Exposure times were typically maintained at 300 ms or 500 ms for microtubules, histones, Dlic and Rod, and at 1000 ms or 1500 ms for kinetochores and centrosomes. Images were processed with Fiji (Schindelin et al, 2012), including analysis of streaming of faint Rod signals with kymographs.

### Introduction of a C3386 codon into human DYNC1H1

Phusion High Fidelity Master Mix with GC buffer (New England Biolabs) was used for site-directed mutagenesis of pDyn1, which contains human DYNC1H1 sequences (accession number NM_001376.4) that are codon optimized for Sf9 insect cell expression. The presence of the mutation encoding the S3386C substitution, as well as the absence of other non-synonymous mutations, was confirmed using Sanger sequencing (Genewiz) and whole-plasmid next generation sequencing (MGH Center for Computational and Integrative Biology DNA Core).

### Production of dynein, dynactin and BICD2N

Wild-type and S3386C-containing human dynein complexes were produced as described (Hoang et al, 2017; Schlager et al, 2014). Briefly, they were expressed recombinantly in Sf9 cells by transposition into the baculovirus genome of sequences encoding wild-type or S3386 DYNC1H1 (tagged with SNAP for fluorescent labelling and ZZ (a synthetic Fc region-binding domain of protein A) for protein purification), as well as the other dynein subunits (DYNC1I2 [DIC2; AF134477], DYNC1LI2 [DLIC2; NM_006141.2], DYNLT1 [Tctex1; NM_006519.2], DYNLL1 [LC8; NM_003746.2] and DYNLRB1 [Robl1; NM_014183.3]). Dynein complexes were captured from Sf9 cell lysates using IgG Sepharose 6 FastFlow beads (GE Healthcare), labeled with SNAP-Cell-TMR-Star, and eluted with Tobacco Etch Virus protease by virtue of a cleavage site between the ZZ and SNAP tags. Dynein complexes were further purified by fast protein liquid chromatography (TSKgel G4000SWxl column [TOSOH Bioscience]) and concentrated by centrifugation through an Amicon Ultra-4 Centrifugal Filter Device (Merck Millipore). Native dynactin was purified from fresh pig brains as previously described (Schlager et al, 2014; Urnavicius et al, 2015) using a series of chromatography steps (XK 50/30 cationic exchange column [GE Healthcare] packed with SP-Sepharose Fast Flow [GE Healthcare], MonoQ HR 16/10 anionic exchange column [GE Healthcare] and TSKgel G4000SWxl column). SNAP-BICD2N and BICD2N-GFP (both containing residues 1-400 of mouse BICD2) were expressed and purified using the Sf9 baculovirus system, as previously described (Belyy et al, 2016; Schlager et al, 2014).

### Assessing motility of isolated dynein-dynactin-BICD2N complexes

Motility assays were performed using established protocols (Hoang et al, 2017; McClintock et al, 2018; Schlager et al, 2014). Briefly, biotin- and HiLyte-488-labeled pig brain microtubules were polymerized *in vitro* using commercial tubulin sources (Cytoskeleton Inc.) and stabilized with taxol (Sigma Aldrich) and GMPCPP (Jena Bioscience) before being adhered to a streptavidin-coated coverslip within the flow chamber. Approximately 30 min before imaging, a dynein-dynactin-BICD2N (DDB) assembly mix was prepared on ice by combining 100 nM TMR-labeled human dynein (wild type or S3386C), 200 nM pig dynactin and 1 μM SNAP-BICD2N in motility buffer (30 mM HEPES pH 7.3, 5 mM MgSO_4_, 1 mM EGTA pH 7.3, 1 mM DTT). A ‘motility mix’ comprising 2.5% (v/v) DDB assembly mix, 2.5 mM MgATP (Sigma Aldrich), 10% (v/v) oxygen scavenging system (1.25 μM glucose oxidase [Sigma Aldrich], 140 nM catalase [Sigma Aldrich], 71 mM β-mercaptoethanol and 25 mM D-glucose) in motility buffer supplemented with 50 mM KCl, 1 mg ml^−1^ α-casein and 20 μM taxol was introduced into the microtubule-containing flow chambers.

TIRF imaging was performed at room temperature (~23 ± 1°C) using a Nikon TIRF system equipped with a back illuminated EMCCD camera iXon^EM+^ DU-897E (Andor) and a 100x/1.49 NA oil-immersion APO TIRF objective, and controlled with μManager software (Edelstein et al, 2010). Three movies were acquired per chamber, with acquisition for 500 frames at the maximum achievable frame rate (~2 frames s^−1^) and 100-ms exposure per frame. Pixel size for each image series was 105 nm x 105 nm.

Analyses of dynein behavior was performed manually using kymographs in Fiji, as described (Hoang et al, 2017). Kymographs were generated from 5 microtubules per movie, followed by tracking of dynein movements with the Multipoint tool. Microtubule binding events were only counting if they lasted for ≥ 1.5 s (3 pixels in the y-axis) and processive events only counted when travel distance was ≥ 500 nm (5 pixels in the x-axis). Because DDB complexes could change speed during runs, mean velocity was calculated from individual velocity segments, as described previously (Schlager et al, 2014). The identities of kymographs were blinded before analysis using the BlindAnalysis macro in ImageJ.

### Optical trap-based force measurements

Optical trapping of DDB complexes was performed as described previously (Belyy et al, 2016). Briefly, DDB complexes were assembled with 1 μl of 0.38 mg ml^−1^ (mutant) or 1 μl 1.06 mg ml^−1^ (wild-type) dynein, 1 μl of 2.25 mg ml^−1^ dynactin, and 1 μl of 1.04 mg ml^−1^ BICD2N-GFP (Belyy et al, 2016) for 10 min at 4°C. The protein mixture was then added to 800-nm diameter carboxylated polystyrene beads that were coated with a rabbit polyclonal anti-GFP antibody (BioLegend MMS-118P), and incubated for 10 min. Flow chambers were decorated with sea urchin axonemes in MB buffer (30 mM HEPES pH 7.0, 5 mM MgSO4, 1 mM EGTA, 10% glycerol). The motor-bead mixture was then introduced to the chamber in imaging buffer (MB supplemented with oxygen scavenging system and 2 mM MgATP). The bead concentration was held constant for all measurements. To ensure that more than ~95% of beads were driven by single dynein motors, the protein mixture was diluted before incubating with beads such that a maximum of 30% of beads exhibited activity when brought into contact with an axoneme (Belyy et al, 2016).

Optical trapping experiments were performed on a custom-built optical trap microscope set-up. DDB-bound beads were trapped with a 2 W 1,064-nm laser beam (Coherent) that was focused on the image plane using a 100x/1.49 N.A. oil-immersion objective (Nikon). Axonemes were located by brightfield imaging and moved to the center of the field-of-view with a locking XY stage (M-687, Physik Instrumente). Trapped beads were lowered to the axoneme surface with a piezo flexure objective scanner (P-721 PIFOC, Physik Instrumente). The position of the bead relative to the trap center was monitored by imaging the back-focal plane of a 1.4 N.A. oil-immersion condenser (Nikon) on a position-sensitive detector (First Sensor). A pair of perpendicular acousto-optical deflectors (AA Opto-Electronic) was used to control beam steering. To calibrate the detector response, a trapped bead was rapidly raster-scanned by the acousto-optical deflector and trap stiffness derived from the Lorentzian fit to the power spectrum of the trapped bead. The laser power was adjusted with a half-wave plate on a motorized rotary mount and maintained at a constant value of 80 mW, corresponding to a spring constant of 0.05 – 0.06 pN nm^−1^. Before data collection for each sample, the spring constant of a trapped bead was recorded.

In the case of DDB complexes formed with wild-type dynein, custom MATLAB software was used to extract stall forces and stall times from raw traces (following down-sampling from 5,000 Hz to 250 Hz). Stall events were defined as a stationary period of a bead at forces larger than 2.5 pN and durations longer than 100 ms, which were followed by snapping back of the bead to the trap center. Stall force was defined as the mean force during the last 20% of the stall event. The stall time was defined as the interval that the bead spent at a force of at least 80% of the stall force. All stall events were plotted and manually reviewed to confirm the accuracy of the computed values. Due to the distinct stall behavior of S3386C dynein, stall forces and stall times of this complex were calculated manually by reviewing each trace for stationary periods of a bead lasting longer than 150 ms at forces larger than 1 pN. The probability of dynein-driven beads sampling different forces under the trap was calculated by combining all the trajectories of wild-type or mutant dynein, plotting the normalized histogram of dynein at binned forces, and fitting the histogram to 3 Gaussians in MATLAB. The peak at near 0 pN represents the time the beads spend unbound from the microtubule. In Figure 9A, this peak was subtracted from the histograms to show the behavior of the beads when they are driven by dynein along the microtubule.

### Molecular dynamic simulations

The mouse cytoplasmic dynein-1 MTBD and partial stalk structure in complex with α/β-tubulin (PDB 6RZB; Lacey et al, 2019) was used to generate a model of the equivalent wild-type human cytoplasmic dynein-1 sequences bound to the tubulin heterodimer. Sequence alignment of the mouse and human MTBDs revealed no sequence divergence. However, two C-to-A mutations (at residues 3323 and 3387) had been introduced in the mouse MTBD used for cryo-EM and these were corrected in the human dynein model using the Mutator plugin of VMD (Humphrey et al, 1996). We used the same plugin to subsequently introduce the S3386C mutation into the model.

α-tubulin residues P37 to D47 are missing in PDB 6RZB, presumably because they are unstructured. A peptide was therefore constructed via the Molefacture plugin of VMD that has P37-D47 as an unstructured region and the flanking regions E23-M36 and S48-E55 as an α-helix and β-sheet, respectively. The constructed peptide was solvated in a water box and 150 mM KCl for MD simulations. The peptide system was minimized for 10,000 steps and followed by 2 ns of equilibration. After equilibration, the peptide was fitted into the remaining tubulin structure via targeted MD simulations (Schlitter et al, 1994) in which the flanking structured regions were slowly (10 ns) pushed into their crystal coordinates. Subsequently, the P37-D47 stretch was incorporated into the remaining tubulin structure using VMD’s merge extension.

MTBD-tubulin systems were aligned with the longitudinal axis of the α/β-tubulin dimer in the z direction. Each system was then solvated in a TIP3P water box with 35 Å cushions in each x-direction (70 Å water cushion in total) to provide enough space for stalk movements; 15 Å cushions were applied in all other directions. Systems were neutralized and ion concentrations set to 150 mM KCl. The size of the solvated system was ~230,000 atoms. Simulations were performed at 310 K and 1 atm pressure with a time step of 2 fs. Langevin dynamics with a damping coefficient of 1 ps^−1^ were used to keep the temperature constant. The pressure was kept at 1 atm using the Langevin Nosé–Hoover method with a 100-fs oscillation period and a 50-fs damping time scale. Long-range electrostatic interactions were calculated with the particle-mesh Ewald method with a cut-off of 12 Å for van der Waals interactions. Prior to running MD simulations, 10,000 steps of minimization followed by 2 ns of equilibration were performed by keeping the proteins fixed. Subsequently, 10,000 steps of minimization were applied without any constraints on dynein and on tubulin, followed by 4 ns of equilibration with 1 kcal mol^−1^ Å^−2^ harmonic constraints on the C_α_. Starting from the equilibrated systems, 900- ns-long production runs were initiated with constraints on R2-H28, F53-E55, H61-D69, P72- R79, L92-S94, F103-N128, G131-N139, G144-Y161, S165-I171, V182-L195, V202-L217, Y224-S241, I252-L259, L269-A273, V288-F296, Y312-G321, P325-T337, F352-V353, R373- S381, R373-S381, and I384-H393 residues of α-tubulin and R2-H28, D41-T55, R58-D67, G71-A78, G82-F92, W101-G126, L130-S138, S145-L151, I163-V169, V180-S188, E198- M200, N204-T214, Y222-R241, G244-L246, L250-N256, F265-A271, V286-M299, Y310- G319, T323-N337, I349-C354, M363-S371, A373-Q391, R384-A393, and L395-G400 residues of β-tubulin to prevent structural deformation of the α/β-tubulin dimer due to the rest of the microtubule being absent. All MD simulations were performed in NAMD-2.14 (Phillips et al, 2020) using the CHARMM36 all-atom additive protein force field (Best et al, 2012).

To calculate MTBD angles in the plane perpendicular to the longitudinal microtubule axis, principal axes (PA) of microtubules were obtained using the VMD Orient tool. All PAs started from the origin located at the center of mass of tubulin and were referenced to the MTBD-tubulin structure. PA1 corresponds to the longitudinal axis of the protofilament. PA2 is the radial axis pointing towards the center of the A3295 and W3395 C_α_ atoms of dynein. PA3 is the tangential axis perpendicular to both PA1 and PA2. The MTBD vector, which indicates the combined MTBD and stalk base orientation with respect to the tubulin, was defined as the vector pointing from the center of dynein’s A3295 and W3395 C_α_ atoms toward the center of its A3288 C_α_ and Y3402 C_α_ atoms. To calculate the MTBD angle, the MTBD vector was projected onto the plane created by PA1 and PA2, and the angle between the projected vector and PA2 was evaluated.

We calculated the stalk angles relative to the microtubule longitudinal axis using the same procedure employed for the MTBD angles, but with a different selection of dynein atoms. Specifically, we superimposed the full-length monomeric dynein structure (PBD, 7Z8F; (Chaaban & Carter, 2022) with the MD conformations by using the C_α_ atoms of the stalk's base, including residues A3295 to W3395 and A3288 C_α_ to Y3402, for alignment. We defined the stalk vector as the vector that originates from the center of dynein’s A3295 and W3395 C_α_ atoms, as sampled in the MD simulations, and extends towards the center of its R3191 C_α_ and S3501 C_α_ atoms within the superimposed structure.

The criteria for determining interactions were as follows. Salt bridge formations were detected using a maximum cut-off distance of 4 Å between the basic nitrogen and acidic oxygens (Barlow & Thornton, 1983). For hydrophobic interactions, a cutoff distance of 8 Å between side chain carbon atoms was used (Manavalan & Ponnuswamy, 1977; Stavrakoudis et al, 2009; Stock et al, 2015). To detect hydrogen bond formation, a maximum 3.5 Å distance between hydrogen bond donor and acceptor and a 30° angle between the hydrogen atom, the donor heavy atom, and the acceptor heavy atom was used (Durrant & McCammon, 2011).

### Statistics and data plotting

Statistical analysis and data plotting was performed with Prism 7.0b (GraphPad). Appropriate statistical tests were selected based on confirmed or assumed data distributions (Gaussian or non-Gaussian), variance (equal or unequal), sample size and number of comparisons (pairwise or multiple). Details of statistical tests applied for each dataset are provided in the relevant figure legends. Violin plots were used instead of bar charts when each group within a dataset had at least 30 values.

## Supplementary Material

Supplement 1

Supplement 2

Supplement 3

Supplement 4

Supplement 5

## References

[R1] Antony D, Brunner HG, Schmidts M (2021) Ciliary dyneins and dynein related ciliopathies. Cells 10. doi: 10.3390/cells10081885 PMC8391580 34440654

[R2] Archambault V, Pinson X (2010) Free centrosomes: where do they all come from? Fly 4: 172–177. doi: 10.4161/fly.4.2.11674 20404481

[R3] Bader JR, Vaughan KT (2010) Dynein at the kinetochore: Timing, interactions and functions. Semin Cell Dev Biol 21: 269–275. doi: 10.1016/j.semcdb.2009.12.015 20045078 PMC2860529

[R4] Barisic M, Maiato H (2015) Dynein prevents erroneous kinetochore-microtubule attachments in mitosis. Cell Cycle 14: 3356–3361. doi: 10.1080/15384101.2015.1089369 26397382 PMC4825607

[R5] Barlow DJ, Thornton JM (1983) Ion-pairs in proteins. J Mol Biol 168: 867–885. doi: 10.1016/s0022-2836(83)80079-5 6887253

[R6] Basto R, Scaerou F, Mische S, Wojcik E, Lefebvre C, Gomes R, Hays T, Karess R (2004) *In vivo* dynamics of the rough deal checkpoint protein during *Drosophila* mitosis. Curr Biol 14: 56–61. doi: 10.1016/j.cub.2003.12.025 14711415

[R7] Belyy V, Schlager MA, Foster H, Reimer AE, Carter AP, Yildiz A (2016) The mammalian dynein-dynactin complex is a strong opponent to kinesin in a tug-of-war competition. Nat Cell Biol 18: 1018–1024. doi: 10.1038/ncb3393 27454819 PMC5007201

[R8] Best RB, Zhu X, Shim J, Lopes PE, Mittal J, Feig M, Mackerell AD Jr., (2012) Optimization of the additive CHARMM all-atom protein force field targeting improved sampling of the backbone ϕ, ψ and side-chain χ1 and χ2 dihedral angles. J Chem Theory Comput 8: 3257–3273. doi: 10.1021/ct300400x 23341755 PMC3549273

[R9] Borgal L, Wakefield JG (2018) Context-dependent spindle pole focusing. Essays Biochem 62: 803–813. doi: 10.1042/EBC20180034 30429281

[R10] Buffin E, Emre D, Karess RE (2007) Flies without a spindle checkpoint. Nat Cell Biol 9: 565–572. doi: 10.1038/ncb1570 17417628

[R11] Catalano C, Al Mughram MH, Guo Y, Kellogg GE (2021) 3D interaction homology: Hydropathic interaction environments of serine and cysteine are strikingly different and their roles adapt in membrane proteins. Curr Res Struct Biol 3: 239–256. doi: 10.1016/j.crstbi.2021.09.002 34693344 PMC8517007

[R12] Chaaban S, Carter AP (2022) Structure of dynein-dynactin on microtubules shows tandem adaptor binding. Nature 610: 212–216. doi: 10.1038/s41586-022-05186-y 36071160 PMC7613678

[R13] Conduit PT, Wainman A, Novak ZA, Weil TT, Raff JW (2015) Re-examining the role of *Drosophila* Sas-4 in centrosome assembly using two-colour-3D-SIM FRAP. eLife 4. doi: 10.7554/eLife.08483 PMC4631861 26530814

[R14] Cross RA, McAinsh A (2014) Prime movers: the mechanochemistry of mitotic kinesins. Nat Rev Mol Cell Biol 15: 257–271. doi: 10.1038/nrm3768 24651543

[R15] Defachelles L, Hainline SG, Menant A, Lee LA, Karess RE (2015) A maternal effect rough deal mutation suggests that multiple pathways regulate *Drosophila* RZZ kinetochore recruitment. J Cell Sci 128: 1204–1216. doi: 10.1242/jcs.165712 25616898 PMC4359924

[R16] Durrant JD, McCammon JA (2011) HBonanza: a computer algorithm for molecular-dynamics-trajectory hydrogen-bond analysis. J Mol Graph Model 31: 5–9. doi: 10.1016/j.jmgm.2011.07.008 21880522 PMC3218086

[R17] Edelstein A, Amodaj N, Hoover K, Vale R, Stuurman N (2010) Computer control of microscopes using μManager. Curr Protoc Mol Biol Chapter 14: Unit14.20. doi: 10.1002/0471142727.mb1420s92 PMC3065365 20890901

[R18] Fahmy K, Akber M, Cai X, Koul A, Hayder A, Baumgartner S (2014) αTubulin 67C and Ncd are essential for establishing a cortical microtubular network and formation of the *Bicoid* mRNA gradient in *Drosophila*. PLoS One 9: e112053. doi: 10.1371/journal.pone.0112053 25390693 PMC4229129

[R19] Firestone AJ, Weinger JS, Maldonado M, Barlan K, Langston LD, O'Donnell M, Gelfand VI, Kapoor TM, Chen JK (2012) Small-molecule inhibitors of the AAA+ ATPase motor cytoplasmic dynein. Nature 484: 125–129. doi: 10.1038/nature10936 22425997 PMC3321072

[R20] Fujimitsu K, Yamano H (2021) Dynamic regulation of mitotic ubiquitin ligase APC/C by coordinated Plx1 kinase and PP2A phosphatase action on a flexible Apc1 loop. EMBO J 40: e107516. doi: 10.15252/embj.2020107516 34291488 PMC8441438

[R21] Fumagalli L, Young FL, Boeynaems S, De Decker M, Mehta AR, Swijsen A, Fazal R, Guo W, Moisse M, Beckers J (2021) C9orf72-derived arginine-containing dipeptide repeats associate with axonal transport machinery and impede microtubule-based motility. Sci Adv 7. doi: 10.1126/sciadv.abg3013 PMC8034861 33837088

[R22] Gaglio T, Dionne MA, Compton DA (1997) Mitotic spindle poles are organized by structural and motor proteins in addition to centrosomes. J Cell Biol 138: 1055–1066. doi: 10.1083/jcb.138.5.1055 9281583 PMC2136753

[R23] Gallaud E, Richard-Parpaillon L, Bataille L, Pascal A, Metivier M, Archambault V, Giet R (2022) The spindle assembly checkpoint and the spatial activation of Polo kinase determine the duration of cell division and prevent tumor formation. PLoS Genet 18: e1010145. doi: 10.1371/journal.pgen.1010145 35377889 PMC9009772

[R24] Gama JB, Pereira C, Simoes PA, Celestino R, Reis RM, Barbosa DJ, Pires HR, Carvalho C, Amorim J, Carvalho AX (2017) Molecular mechanism of dynein recruitment to kinetochores by the Rod-Zw10-Zwilch complex and Spindly. J Cell Biol 216: 943–960. doi: 10.1083/jcb.201610108 28320824 PMC5379953

[R25] Gassmann R (2023) Dynein at the kinetochore. J Cell Sci 136. doi: 10.1242/jcs.220269 36861883

[R26] Gassmann R, Essex A, Hu JS, Maddox PS, Motegi F, Sugimoto A, O'Rourke SM, Bowerman B, McLeod I, Yates JR 3rd, (2008) A new mechanism controlling kinetochore-microtubule interactions revealed by comparison of two dynein-targeting components: SPDL-1 and the Rod/Zwilch/Zw10 complex. Genes Dev 22: 2385–2399. doi: 10.1101/gad.1687508 18765790 PMC2532926

[R27] Gassmann R, Holland AJ, Varma D, Wan X, Civril F, Cleveland DW, Oegema K, Salmon ED, Desai A (2010) Removal of Spindly from microtubule-attached kinetochores controls spindle checkpoint silencing in human cells. Genes Dev 24: 957–971. doi: 10.1101/gad.1886810 20439434 PMC2861194

[R28] Gepner J, Li M, Ludmann S, Kortas C, Boylan K, Iyadurai SJ, McGrail M, Hays TS (1996) Cytoplasmic dynein function is essential in *Drosophila melanogaster*. Genetics 142: 865–878. doi: 10.1093/genetics/142.3.865 8849893 PMC1207024

[R29] Goddard TD, Huang CC, Meng EC, Pettersen EF, Couch GS, Morris JH, Ferrin TE (2018) UCSF ChimeraX: Meeting modern challenges in visualization and analysis. Protein Sci 27: 14–25. doi: 10.1002/pro.3235 28710774 PMC5734306

[R30] Gonczy P, Pichler S, Kirkham M, Hyman AA (1999) Cytoplasmic dynein is required for distinct aspects of MTOC positioning, including centrosome separation, in the one cell stage *Caenorhabditis elegans* embryo. J Cell Biol 147: 135–150. doi: 10.1083/jcb.147.1.135 10508861 PMC2164971

[R31] Griffis ER, Stuurman N, Vale RD (2007) Spindly, a novel protein essential for silencing the spindle assembly checkpoint, recruits dynein to the kinetochore. J Cell Biol 177: 1005–1015. doi: 10.1083/jcb.200702062 17576797 PMC2064361

[R32] Hafezparast M, Klocke R, Ruhrberg C, Marquardt A, Ahmad-Annuar A, Bowen S, Lalli G, Witherden AS, Hummerich H, Nicholson S (2003) Mutations in dynein link motor neuron degeneration to defects in retrograde transport. Science 300: 808–812. doi: 10.1126/science.1083129 12730604

[R33] Harada A, Takei Y, Kanai Y, Tanaka Y, Nonaka S, Hirokawa N (1998) Golgi vesiculation and lysosome dispersion in cells lacking cytoplasmic dynein. J Cell Biol 141: 51–59. doi: 10.1083/jcb.141.1.51 9531547 PMC2132725

[R34] Hartenstein V, Younossi-Hartenstein A, Lovick JK, Kong A, Omoto JJ, Ngo KT, Viktorin G (2015) Lineage-associated tracts defining the anatomy of the *Drosophila* first instar larval brain. Dev Biol 406: 14–39. doi: 10.1016/j.ydbio.2015.06.021 26141956 PMC4936782

[R35] Heeger S, Leismann O, Schittenhelm R, Schraidt O, Heidmann S, Lehner CF (2005) Genetic interactions of separase regulatory subunits reveal the diverged *Drosophila* Cenp-C homolog. Genes Dev 19: 2041–2053. doi: 10.1101/gad.347805 16140985 PMC1199574

[R36] Hinchcliffe EH, Vaughan KT (2017) Cytoplasmic dynein during mitosis. In Dyneins: The biology of dynein motors, King SM (ed), Second edn.: Elsevier

[R37] Hirokawa N, Noda Y, Tanaka Y, Niwa S (2009) Kinesin superfamily motor proteins and intracellular transport. Nat Rev Mol Cell Biol 10: 682–696. doi: 10.1038/nrm2774 19773780

[R38] Hoang HT, Schlager MA, Carter AP, Bullock SL (2017) DYNC1H1 mutations associated with neurological diseases compromise processivity of dynein-dynactin-cargo adaptor complexes. Proc Natl Acad Sci U S A 114: E1597–E1606. doi: 10.1073/pnas.1620141114 28196890 PMC5338514

[R39] Hoing S, Yeh TY, Baumann M, Martinez NE, Habenberger P, Kremer L, Drexler HCA, Kuchler P, Reinhardt P, Choidas A (2018) Dynarrestin, a novel inhibitor of cytoplasmic dynein. Cell Chem Biol 25: 357–369 e356. doi: 10.1016/j.chembiol.2017.12.014 29396292 PMC8543760

[R40] Howell BJ, McEwen BF, Canman JC, Hoffman DB, Farrar EM, Rieder CL, Salmon ED (2001) Cytoplasmic dynein/dynactin drives kinetochore protein transport to the spindle poles and has a role in mitotic spindle checkpoint inactivation. J Cell Biol 155: 1159–1172. doi: 10.1083/jcb.200105093 11756470 PMC2199338

[R41] Hu Y, Sopko R, Chung V, Foos M, Studer RA, Landry SD, Liu D, Rabinow L, Gnad F, Beltrao P (2019) iProteinDB: An integrative database of *Drosophila* post-translational modifications. G3 9: 1–11. doi: 10.1534/g3.118.200637 30397019 PMC6325894

[R42] Huang J, Raff JW (1999) The disappearance of cyclin B at the end of mitosis is regulated spatially in *Drosophila* cells. EMBO J 18: 2184–2195. doi: 10.1093/emboj/18.8.2184 10205172 PMC1171302

[R43] Humphrey W, Dalke A, Schulten K (1996) VMD: visual molecular dynamics. J Mol Graph 14: 33-38, 27–38. doi: 10.1016/0263-7855(96)00018-5 8744570

[R44] Hurd DD, Saxton WM (1996) Kinesin mutations cause motor neuron disease phenotypes by disrupting fast axonal transport in *Drosophila*. Genetics 144: 1075–1085. doi: 10.1093/genetics/144.3.1075 8913751 PMC1207603

[R45] Iyadurai SJ, Li MG, Gilbert SP, Hays TS (1999) Evidence for cooperative interactions between the two motor domains of cytoplasmic dynein. Curr Biol 9: 771–774. doi: 10.1016/s0960-9822(99)80340-6 10421581

[R46] Jumper J, Evans R, Pritzel A, Green T, Figurnov M, Ronneberger O, Tunyasuvunakool K, Bates R, Zidek A, Potapenko A (2021) Highly accurate protein structure prediction with AlphaFold. Nature 596: 583–589. doi: 10.1038/s41586-021-03819-2 34265844 PMC8371605

[R47] Krenn V, Musacchio A (2015) The Aurora B kinase in chromosome bi-orientation and spindle checkpoint signaling. Front Oncol 5: 225. doi: 10.3389/fonc.2015.00225 26528436 PMC4607871

[R48] Laan L, Roth S, Dogterom M (2012) End-on microtubule-dynein interactions and pulling-based positioning of microtubule organizing centers. Cell Cycle 11: 3750–3757. doi: 10.4161/cc.21753 22895049 PMC3495818

[R49] Lacey SE, He S, Scheres SH, Carter AP (2019) Cryo-EM of dynein microtubule-binding domains shows how an axonemal dynein distorts the microtubule. eLife 8. doi: 10.7554/eLife.47145 PMC6629372 31264960

[R50] Li Y, Yu W, Liang Y, Zhu X (2007) Kinetochore dynein generates a poleward pulling force to facilitate congression and full chromosome alignment. Cell Res 17: 701–712. doi: 10.1038/cr.2007.65 17680027

[R51] Liu D, Davydenko O, Lampson MA (2012) Polo-like kinase-1 regulates kinetochore-microtubule dynamics and spindle checkpoint silencing. J Cell Biol 198: 491–499. doi: 10.1083/jcb.201205090 22908307 PMC3514039

[R52] Logarinho E, Bousbaa H, Dias JM, Lopes C, Amorim I, Antunes-Martins A, Sunkel CE (2004) Different spindle checkpoint proteins monitor microtubule attachment and tension at kinetochores in *Drosophila* cells. J Cell Sci 117: 1757–1771. doi: 10.1242/jcs.01033 15075237

[R53] Loppin B, Dubruille R, Horard B (2015) The intimate genetics of *Drosophila* fertilization. Open Biol 5. doi: 10.1098/rsob.150076 PMC4554920 26246493

[R54] Lu W, Gelfand VI (2017) Moonlighting motors: Kinesin, dynein, and cell polarity. Trends Cell Biol 27: 505–514. doi: 10.1016/j.tcb.2017.02.005 28284467 PMC5476484

[R55] Lucas EP, Raff JW (2007) Maintaining the proper connection between the centrioles and the pericentriolar matrix requires *Drosophila* centrosomin. J Cell Biol 178: 725–732. doi: 10.1083/jcb.200704081 17709428 PMC2064537

[R56] Maddox P, Desai A, Oegema K, Mitchison TJ, Salmon ED (2002) Poleward microtubule flux is a major component of spindle dynamics and Anaphase a in mitotic *Drosophila* embryos. Curr Biol 12: 1670–1674. doi: 10.1016/s0960-9822(02)01183-1 12361570

[R57] Manavalan P, Ponnuswamy PK (1977) A study of the preferred environment of amino acid residues in globular proteins. Arch Biochem Biophys 184: 476–487. doi: 10.1016/0003-9861(77)90457-x 596883

[R58] Maresca TJ, Salmon ED (2010) Welcome to a new kind of tension: translating kinetochore mechanics into a wait-anaphase signal. J Cell Sci 123: 825–835. doi: 10.1242/jcs.064790 20200228 PMC2831757

[R59] Martin M, Iyadurai SJ, Gassman A, Gindhart JG Jr., Hays TS, Saxton WM (1999) Cytoplasmic dynein, the dynactin complex, and kinesin are interdependent and essential for fast axonal transport. Mol Biol Cell 10: 3717–3728. doi: 10.1091/mbc.10.11.3717 10564267 PMC25669

[R60] Marzo MG, Griswold JM, Ruff KM, Buchmeier RE, Fees CP, Markus SM (2019) Molecular basis for dyneinopathies reveals insight into dynein regulation and dysfunction. eLife 8. doi: 10.7554/eLife.47246 PMC6733598 31364990

[R61] Mathe E, Boros I, Josvay K, Li K, Puro J, Kaufman TC, Szabad J (1998) The *Tomaj* mutant alleles of αTubulin67C reveal a requirement for the encoded maternal specific tubulin isoform in the sperm aster, the cleavage spindle apparatus and neurogenesis during embryonic development in *Drosophila*. J Cell Sci 111 (Pt 7): 887–896. doi: 10.1242/jcs.111.7.887 9490633

[R62] Matthews KA, Miller DF, Kaufman TC (1989) Developmental distribution of RNA and protein products of the *Drosophila* α-tubulin gene family. Dev Biol 132: 45–61. doi: 10.1016/0012-1606(89)90203-0 2492961

[R63] Matthews KA, Rees D, Kaufman TC (1993) A functionally specialized α-tubulin is required for oocyte meiosis and cleavage mitoses in *Drosophila*. Development 117: 977–991. doi: 10.1242/dev.117.3.977 8325246

[R64] McCleland ML, O'Farrell PH (2008) RNAi of mitotic cyclins in *Drosophila* uncouples the nuclear and centrosome cycle. Curr Biol 18: 245–254. doi: 10.1016/j.cub.2008.01.041 18291653 PMC2698964

[R65] McClintock MA, Dix CI, Johnson CM, McLaughlin SH, Maizels RJ, Hoang HT, Bullock SL (2018) RNA-directed activation of cytoplasmic dynein-1 in reconstituted transport RNPs. eLife 7. doi: 10.7554/eLife.36312 PMC6056234 29944118

[R66] McKenney RJ, Huynh W, Tanenbaum ME, Bhabha G, Vale RD (2014) Activation of cytoplasmic dynein motility by dynactin-cargo adapter complexes. Science 345: 337–341. doi: 10.1126/science.1254198 25035494 PMC4224444

[R67] McVey SL, Cosby JK, Nannas NJ (2021) Aurora B tension sensing mechanisms in the kinetochore ensure accurate chromosome segregation. Int J Mol Sci 22. doi: 10.3390/ijms22168818 PMC8396173 34445523

[R68] Melkov A, Baskar R, Alcalay Y, Abdu U (2016) A new mode of mitochondrial transport and polarized sorting regulated by Dynein, Milton and Miro. Development 143: 4203–4213. doi: 10.1242/dev.138289 27707795

[R69] Merdes A, Ramyar K, Vechio JD, Cleveland DW (1996) A complex of NuMA and cytoplasmic dynein is essential for mitotic spindle assembly. Cell 87: 447–458. doi: 10.1016/s0092-8674(00)81365-3 8898198

[R70] Miller KE, DeProto J, Kaufmann N, Patel BN, Duckworth A, Van Vactor D (2005) Direct observation demonstrates that Liprin-α is required for trafficking of synaptic vesicles. Curr Biol 15: 684–689. doi: 10.1016/j.cub.2005.02.061 15823543

[R71] Mirdita M, Schutze K, Moriwaki Y, Heo L, Ovchinnikov S, Steinegger M (2022) ColabFold: making protein folding accessible to all. Nat Methods 19: 679–682. doi: 10.1038/s41592-022-01488-1 35637307 PMC9184281

[R72] Mirdita M, Steinegger M, Soding J (2019) MMseqs2 desktop and local web server app for fast, interactive sequence searches. Bioinformatics 35: 2856–2858. doi: 10.1093/bioinformatics/bty1057 30615063 PMC6691333

[R73] Mische S, He Y, Ma L, Li M, Serr M, Hays TS (2008) Dynein light intermediate chain: an essential subunit that contributes to spindle checkpoint inactivation. Mol Biol Cell 19: 4918–4929. doi: 10.1091/mbc.E08-05-0483 18799620 PMC2575169

[R74] Morin X, Daneman R, Zavortink M, Chia W (2001) A protein trap strategy to detect GFP-tagged proteins expressed from their endogenous loci in *Drosophila*. Proc Natl Acad Sci U S A 98: 15050–15055. doi: 10.1073/pnas.261408198 11742088 PMC64981

[R75] Mosalaganti S, Keller J, Altenfeld A, Winzker M, Rombaut P, Saur M, Petrovic A, Wehenkel A, Wohlgemuth S, Muller F (2017) Structure of the RZZ complex and molecular basis of its interaction with Spindly. J Cell Biol 216: 961–981. doi: 10.1083/jcb.201611060 28320825 PMC5379955

[R76] Murray A (1995) Cyclin ubiquitination: the destructive end of mitosis. Cell 81: 149–152. doi: 10.1016/0092-8674(95)90322-4 7736567

[R77] Musacchio A (2015) The molecular biology of spindle assembly checkpoint signaling dynamics. Curr Biol 25: R1002–1018. doi: 10.1016/j.cub.2015.08.051 26485365

[R78] Nielsen MG, Gadagkar SR, Gutzwiller L (2010) Tubulin evolution in insects: gene duplication and subfunctionalization provide specialized isoforms in a functionally constrained gene family. BMC Evol Biol 10: 113. doi: 10.1186/1471-2148-10-113 20423510 PMC2880298

[R79] Olenick MA, Holzbaur ELF (2019) Dynein activators and adaptors at a glance. J Cell Sci 132. doi: 10.1242/jcs.227132 PMC6451413 30877148

[R80] Ori-McKenney KM, Xu J, Gross SP, Vallee RB (2010) A cytoplasmic dynein tail mutation impairs motor processivity. Nat Cell Biol 12: 1228–1234. doi: 10.1038/ncb2127 21102439 PMC3385513

[R81] Pandey R, Heeger S, Lehner CF (2007) Rapid effects of acute anoxia on spindle kinetochore interactions activate the mitotic spindle checkpoint. J Cell Sci 120: 2807–2818. doi: 10.1242/jcs.007690 17652159

[R82] Petrova B, Liu K, Tian C, Kitaoka M, Freinkman E, Yang J, Orr-Weaver TL (2018) Dynamic redox balance directs the oocyte-to-embryo transition via developmentally controlled reactive cysteine changes. Proc Natl Acad Sci U S A 115: E7978–E7986. doi: 10.1073/pnas.1807918115 30082411 PMC6112717

[R83] Phillips JC, Hardy DJ, Maia JDC, Stone JE, Ribeiro JV, Bernardi RC, Buch R, Fiorin G, Henin J, Jiang W (2020) Scalable molecular dynamics on CPU and GPU architectures with NAMD. J Chem Phys 153: 044130. doi: 10.1063/5.0014475 32752662 PMC7395834

[R84] Pilling AD, Horiuchi D, Lively CM, Saxton WM (2006) Kinesin-1 and dynein are the primary motors for fast transport of mitochondria in *Drosophila* motor axons. Mol Biol Cell 17: 2057–2068. doi: 10.1091/mbc.e05-06-0526 16467387 PMC1415296

[R85] Poirier K, Lebrun N, Broix L, Tian G, Saillour Y, Boscheron C, Parrini E, Valence S, Pierre BS, Oger M (2013) Mutations in *TUBG1*, *DYNC1H1*, *KIF5C* and *KIF2A* cause malformations of cortical development and microcephaly. Nat Genet 45: 639–647. doi: 10.1038/ng.2613 23603762 PMC3826256

[R86] Port F, Bullock SL (2016) Creating heritable mutations in *Drosophila* with CRISPR-Cas9. Methods Mol Biol 1478: 145–160. doi: 10.1007/978-1-4939-6371-3_7 27730579

[R87] Port F, Chen HM, Lee T, Bullock SL (2014) Optimized CRISPR/Cas tools for efficient germline and somatic genome engineering in *Drosophila*. Proc Natl Acad Sci U S A 111: E2967–2976. doi: 10.1073/pnas.1405500111 25002478 PMC4115528

[R88] Raaijmakers JA, van Heesbeen RG, Meaders JL, Geers EF, Fernandez-Garcia B, Medema RH, Tanenbaum ME (2012) Nuclear envelope-associated dynein drives prophase centrosome separation and enables Eg5-independent bipolar spindle formation. EMBO J 31: 4179–4190. doi: 10.1038/emboj.2012.272 23034402 PMC3492733

[R89] Raff JW, Jeffers K, Huang JY (2002) The roles of Fzy/Cdc20 and Fzr/Cdh1 in regulating the destruction of cyclin B in space and time. J Cell Biol 157: 1139–1149. doi: 10.1083/jcb.200203035 12082076 PMC2173543

[R90] Reck-Peterson SL, Redwine WB, Vale RD, Carter AP (2018) The cytoplasmic dynein transport machinery and its many cargoes. Nat Rev Mol Cell Biol 19: 382–398. doi: 10.1038/s41580-018-0004-3 29662141 PMC6457270

[R91] Robert X, Gouet P (2014) Deciphering key features in protein structures with the new ENDscript server. Nucleic Acids Res 42: W320–324. doi: 10.1093/nar/gku316 24753421 PMC4086106

[R92] Robinson JT, Wojcik EJ, Sanders MA, McGrail M, Hays TS (1999) Cytoplasmic dynein is required for the nuclear attachment and migration of centrosomes during mitosis in *Drosophila*. J Cell Biol 146: 597–608. doi: 10.1083/jcb.146.3.597 10444068 PMC2150560

[R93] Salina D, Bodoor K, Eckley DM, Schroer TA, Rattner JB, Burke B (2002) Cytoplasmic dynein as a facilitator of nuclear envelope breakdown. Cell 108: 97–107. doi: 10.1016/s0092-8674(01)00628-6 11792324

[R94] Savoian MS, Goldberg ML, Rieder CL (2000) The rate of poleward chromosome motion is attenuated in *Drosophila zw10* and *rod* mutants. Nat Cell Biol 2: 948–952. doi: 10.1038/35046605 11146661

[R95] Schiavo G, Greensmith L, Hafezparast M, Fisher EM (2013) Cytoplasmic dynein heavy chain: the servant of many masters. Trends Neurosci 36: 641–651. doi: 10.1016/j.tins.2013.08.001 24035135 PMC3824068

[R96] Schindelin J, Arganda-Carreras I, Frise E, Kaynig V, Longair M, Pietzsch T, Preibisch S, Rueden C, Saalfeld S, Schmid B (2012) Fiji: an open-source platform for biological-image analysis. Nat Methods 9: 676–682. doi: 10.1038/nmeth.2019 22743772 PMC3855844

[R97] Schittenhelm RB, Heeger S, Althoff F, Walter A, Heidmann S, Mechtler K, Lehner CF (2007) Spatial organization of a ubiquitous eukaryotic kinetochore protein network in *Drosophila* chromosomes. Chromosoma 116: 385–402. doi: 10.1007/s00412-007-0103-y 17333235 PMC1950589

[R98] Schlager MA, Hoang HT, Urnavicius L, Bullock SL, Carter AP (2014) *In vitro* reconstitution of a highly processive recombinant human dynein complex. EMBO J 33: 1855–1868. doi: 10.15252/embj.201488792 24986880 PMC4158905

[R99] Schlitter J, Engels M, Kruger P (1994) Targeted molecular dynamics: a new approach for searching pathways of conformational transitions. J Mol Graph 12: 84–89. doi: 10.1016/0263-7855(94)80072-3 7918256

[R100] Schmidt H, Carter AP (2016) Review: Structure and mechanism of the dynein motor ATPase. Biopolymers 105: 557–567. doi: 10.1002/bip.22856 27062277 PMC4879348

[R101] Schneider CA, Rasband WS, Eliceiri KW (2012) NIH Image to ImageJ: 25 years of image analysis. Nat Methods 9: 671–675. doi: 10.1038/nmeth.2089 22930834 PMC5554542

[R102] Sharp DJ, Brown HM, Kwon M, Rogers GC, Holland G, Scholey JM (2000) Functional coordination of three mitotic motors in *Drosophila* embryos. Mol Biol Cell 11: 241–253. doi: 10.1091/mbc.11.1.241 10637305 PMC14771

[R103] Siller KH, Serr M, Steward R, Hays TS, Doe CQ (2005) Live imaging of *Drosophila* brain neuroblasts reveals a role for Lis1/dynactin in spindle assembly and mitotic checkpoint control. Mol Biol Cell 16: 5127–5140. doi: 10.1091/mbc.e05-04-0338 16107559 PMC1266413

[R104] Sivagurunathan S, Schnittker RR, Nandini S, Plamann MD, King SJ (2012) A mouse neurodegenerative Dynein heavy chain mutation alters dynein motility and localization in *Neurospora crassa*. Cytoskeleton 69: 613–624. doi: 10.1002/cm.21049 22991199 PMC3763949

[R105] Sivaram MV, Wadzinski TL, Redick SD, Manna T, Doxsey SJ (2009) Dynein light intermediate chain 1 is required for progress through the spindle assembly checkpoint. EMBO J 28: 902–914. doi: 10.1038/emboj.2009.38 19229290 PMC2670866

[R106] Stavrakoudis A, Tsoulos IG, Shenkarev ZO, Ovchinnikova TV (2009) Molecular dynamics simulation of antimicrobial peptide arenicin-2: β-hairpin stabilization by noncovalent interactions. Biopolymers 92: 143–155. doi: 10.1002/bip.21149 19189382

[R107] Steinman JB, Santarossa CC, Miller RM, Yu LS, Serpinskaya AS, Furukawa H, Morimoto S, Tanaka Y, Nishitani M, Asano M (2017) Chemical structure-guided design of dynapyrazoles, cell-permeable dynein inhibitors with a unique mode of action. eLife 6. doi: 10.7554/eLife.25174 PMC5478271 28524820

[R108] Stock P, Utzig T, Valtiner M (2015) Direct and quantitative AFM measurements of the concentration and temperature dependence of the hydrophobic force law at nanoscopic contacts. J Colloid Interface Sci 446: 244–251. doi: 10.1016/j.jcis.2015.01.032 25678158

[R109] Torroja L, Chu H, Kotovsky I, White K (1999) Neuronal overexpression of APPL, the Drosophila homologue of the amyloid precursor protein (APP), disrupts axonal transport. Curr Biol 9: 489–492. doi: 10.1016/s0960-9822(99)80215-2 10322116

[R110] Urnavicius L, Zhang K, Diamant AG, Motz C, Schlager MA, Yu M, Patel NA, Robinson CV, Carter AP (2015) The structure of the dynactin complex and its interaction with dynein. Science 347: 1441–1446. doi: 10.1126/science.aaa4080 25814576 PMC4413427

[R111] Vagnoni A, Bullock SL (2016) A simple method for imaging axonal transport in aging neurons using the adult *Drosophila* wing. Nat Protoc 11: 1711–1723. doi: 10.1038/nprot.2016.112 27560175 PMC5027916

[R112] Vagnoni A, Hoffmann PC, Bullock SL (2016) Reducing Lissencephaly-1 levels augments mitochondrial transport and has a protective effect in adult *Drosophila* neurons. J Cell Sci 129: 178–190. doi: 10.1242/jcs.179184 26598558 PMC4732301

[R113] Vaisberg EA, Koonce MP, McIntosh JR (1993) Cytoplasmic dynein plays a role in mammalian mitotic spindle formation. J Cell Biol 123: 849–858. doi: 10.1083/jcb.123.4.849 8227145 PMC2200153

[R114] van Heesbeen RG, Tanenbaum ME, Medema RH (2014) Balanced activity of three mitotic motors is required for bipolar spindle assembly and chromosome segregation. Cell Rep 8: 948–956. doi: 10.1016/j.celrep.2014.07.015 25127142

[R115] Varma D, Monzo P, Stehman SA, Vallee RB (2008) Direct role of dynein motor in stable kinetochore-microtubule attachment, orientation, and alignment. J Cell Biol 182: 1045–1054. doi: 10.1083/jcb.200710106 18809721 PMC2542467

[R116] Vazquez-Pianzola P, Beuchle D, Saro G, Hernandez G, Maldonado G, Brunssen D, Meister P, Suter B (2022) Female meiosis II and pronuclear fusion require the microtubule transport factor Bicaudal D. Development 149. doi: 10.1242/dev.199944 35723263

[R117] Venkei Z, Gaspar I, Toth G, Szabad J (2006) α4-Tubulin is involved in rapid formation of long microtubules to push apart the daughter centrosomes during early *Drosophila* embryogenesis. J Cell Sci 119: 3238–3248. doi: 10.1242/jcs.03039 16847053

[R118] Vissers LE, de Ligt J, Gilissen C, Janssen I, Steehouwer M, de Vries P, van Lier B, Arts P, Wieskamp N, del Rosario M (2010) A *de novo* paradigm for mental retardation. Nat Genet 42: 1109–1112. doi: 10.1038/ng.712 21076407

[R119] Vukusic K, Buda R, Tolic IM (2019) Force-generating mechanisms of anaphase in human cells. J Cell Sci 132. doi: 10.1242/jcs.231985 31527150

[R120] West RJ, Lu Y, Marie B, Gao FB, Sweeney ST (2015) Rab8, POSH, and TAK1 regulate synaptic growth in a *Drosophila* model of frontotemporal dementia. J Cell Biol 208: 931–947. doi: 10.1083/jcb.201404066 25800055 PMC4384727

[R121] Whitfield WG, Gonzalez C, Maldonado-Codina G, Glover DM (1990) The A- and B-type cyclins of Drosophila are accumulated and destroyed in temporally distinct events that define separable phases of the G2-M transition. EMBO J 9: 2563–2572. doi: 10.1002/j.1460-2075.1990.tb07437.x 2142452 PMC552287

[R122] Wilkie GS, Davis I (2001) *Drosophila wingless* and pair-rule transcripts localize apically by dynein-mediated transport of RNA particles. Cell 105: 209–219. doi: 10.1016/s0092-8674(01)00312-9 11336671

[R123] Willemsen MH, Vissers LE, Willemsen MA, van Bon BW, Kroes T, de Ligt J, de Vries BB, Schoots J, Lugtenberg D, Hamel BC (2012) Mutations in DYNC1H1 cause severe intellectual disability with neuronal migration defects. J Med Genet 49: 179–183. doi: 10.1136/jmedgenet-2011-100542 22368300

[R124] Wojcik E, Basto R, Serr M, Scaerou F, Karess R, Hays T (2001) Kinetochore dynein: its dynamics and role in the transport of the Rough deal checkpoint protein. Nat Cell Biol 3: 1001–1007. doi: 10.1038/ncb1101-1001 11715021

[R125] Yang Z, Tulu US, Wadsworth P, Rieder CL (2007) Kinetochore dynein is required for chromosome motion and congression independent of the spindle checkpoint. Curr Biol 17: 973–980. doi: 10.1016/j.cub.2007.04.056 17509882 PMC2570756

[R126] Yi JY, Ori-McKenney KM, McKenney RJ, Vershinin M, Gross SP, Vallee RB (2011) High-resolution imaging reveals indirect coordination of opposite motors and a role for LIS1 in high-load axonal transport. J Cell Biol 195: 193–201. doi: 10.1083/jcb.201104076 22006948 PMC3198168

