# Supplementary material for: A force-sensitive mutation reveals a spindle assembly checkpoint-independent role for dynein in anaphase progression
Source: bioRxiv. 2023 Aug 4:2023.08.03.551815. Preprint. [Version 1] doi: 10.1101/2023.08.03.551815 (PMC10418259; doi:10.1101/2023.08.03.551815)
Supplement: Supplement 5 [file NIHPP2023.08.03.551815v1-supplement-5.pdf]

bioRxiv preprint doi: https://doi.org/10.1101/2023.08.03.551815; this version posted August 4, 2023. The copyright holder for this preprint (which was not certified by peer review) is the author/funder, who has granted bioRxiv a license to display the preprint in perpetuity. It is made available under aCC-BY 4.0 International license.

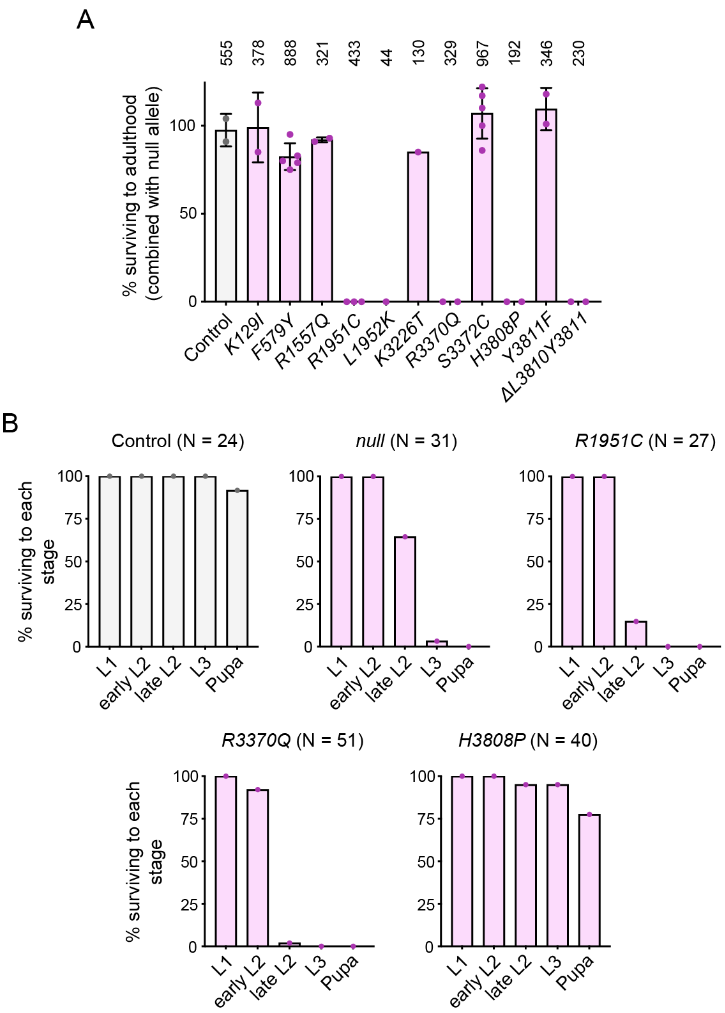

Figure S1. Lethality analysis of Dhc mutations. (A) Complementation tests showing percentage survival to adulthood of flies that are *trans*-heterozygous for the indicated *Dhc* mutation and a Dhc^{null} allele. Columns display mean values from individual crosses; error bars are S.D.; circles are values for individual crosses. Data were normalized based on number of each genotype expected, if there were no lethality, given the total number of offspring. Numbers above each column show total number of offspring assessed for each genotype. (B) Percentage of L1 larvae of indicated genotypes reaching the specified stage (note that there was no overt lethality at the embryonic stage). N is number of animals analyzed for each genotype. In A and B, control animals were *trans*-heterozygous for a wild-type *Dhc* allele (recovered from the same CRISPR-Cas9 mutagenesis experiment that generated the *Dhc* mutant alleles) and the Dhc^{null} allele.

bioRxiv preprint doi: https://doi.org/10.1101/2023.08.03.551815; this version posted August 4, 2023. The copyright holder for this preprint (which was not certified by peer review) is the author/funder, who has granted bioRxiv a license to display the preprint in perpetuity. It is made available under aCC-BY 4.0 International license.

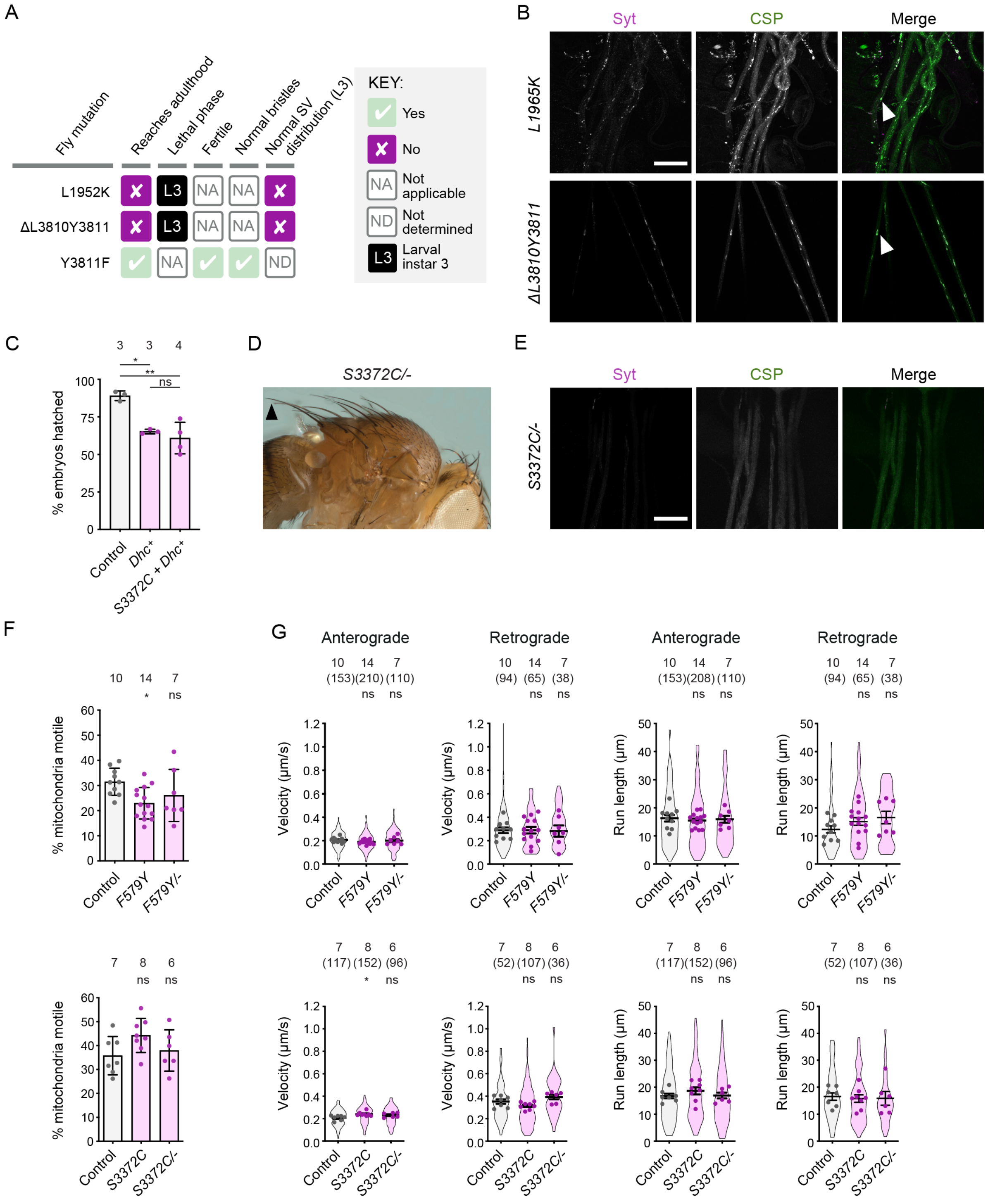

Figure S2. Supplementary data from phenotypic analysis of novel Dhc mutations. *Legend overleaf.*

bioRxiv preprint doi: https://doi.org/10.1101/2023.08.03.551815; this version posted August 4, 2023. The copyright holder for this preprint (which was not certified by peer review) is the author/funder, who has granted bioRxiv a license to display the preprint in perpetuity. It is made available under aCC-BY 4.0 International license.

Figure S2. Supplementary data from phenotypic analysis of novel Dhc mutations. (A) Summary of *in vivo* effects of L1952K, ΔL3810Y3811 and Y3811F. SV, synaptic vesicle; L3, larval instar 3. (B) Confocal images of segmental nerves (taken proximal to the ventral ganglion; anterior to the top; Z-projection) from L3 larvae stained for the synaptic vesicle proteins Synaptotagmin (Syt) and Cysteine-string protein (CSP). Arrowheads show examples of synaptic vesicle accumulations in mutants. Images are representative of 3 – 6 larvae analyzed per genotype. See Figure 1E for images from control. (C) Quantification of hatching rate of eggs laid by mated females of indicated genotypes. Columns show mean values per egg collection; error bars represent S.D.; circles are values for individual egg collections. Number of collections per genotype (each from an independent cross; 414 – 1107 eggs per collection) is shown above bars. The control genotype was *yw*. *Dhc*$^{+}$ is a genomic rescue construct. Note that this construct reduces hatching rate in the wild-type background and that fertility defects of *S3372C* mothers are only suppressed by the transgene to this point. (D) Image (representative of >160 flies examined) showing normal bristles in *S3372C/-* adult flies. Arrowhead points to posterior scutellar macrochaetae. See Figure 1D for control image. (E) Confocal images of segmental nerves (taken proximal to the ventral ganglion; anterior to the top; Z-projection) from fixed L3 larvae stained for Syt and CSP, showing lack of abnormal synaptic vesicle accumulations. Images are representative of 3 larvae analyzed. See Figure 1E for images from control. (F) Quantification of percentage of mitochondria that exhibit transport in any direction in the adult wing nerve during the 3 minutes of data acquisition. Columns show mean values per movie; errors bars represent S.D.; circles are values for individual movies, each from a different wing. Number of wings analyzed is shown above bars. (G) Quantification of velocity and run length of transported mitochondria in the adult wing nerve. Violin plots show values for individual mitochondria and circles show mean values per wing. Horizontal lines shown mean ± S.D. of values for individual wings. Numbers without parentheses above bars are number of wings, with numbers of mitochondria given in parentheses. Evaluation of statistical significance (compared to control) in C, F and G was performed with a 1-way ANOVA with Dunnett's multiple comparisons test (in G, the mean values per wing were compared): **, $P<0.01$; *, $P<0.05$; ns, not significant. Scale bars: B and E, 50 µm.

bioRxiv preprint doi: https://doi.org/10.1101/2023.08.03.551815; this version posted August 4, 2023. The copyright holder for this preprint (which was not certified by peer review) is the author/funder, who has granted bioRxiv a license to display the preprint in perpetuity. It is made available under aCC-BY 4.0 International license.

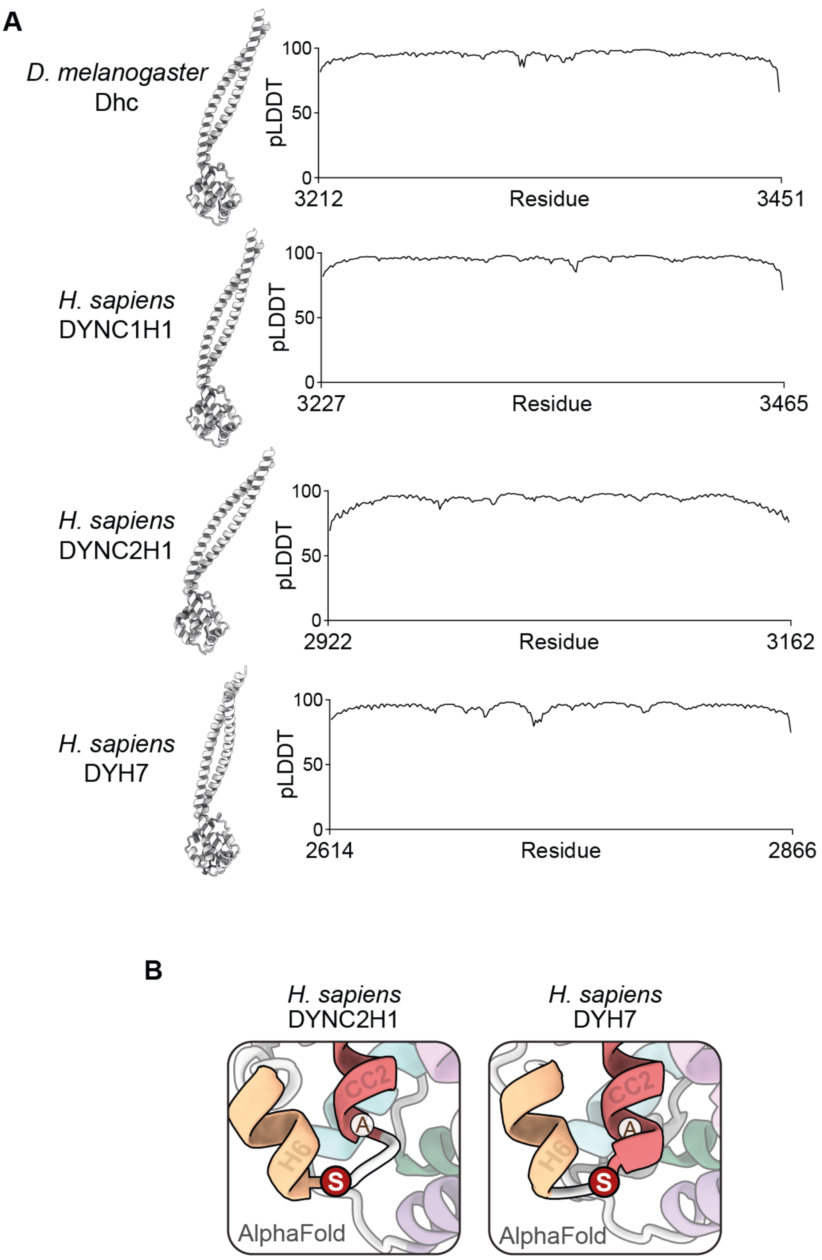

Figure S3. Supplementary data from structural analysis of S3372 in *Drosophila* Dhc and equivalent residues in other dynein family members. (A) Structural overview and pLDDT (predicted Local Distance Difference Test) plots (Jumper *et al*, 2021) of Alphafold2-generated structures of dynein MTBDs and stalks (showing that predictions are high confidence). (B) Zoom ins of regions contain serine residues equivalent to *Drosophila* S3372 in Alphafold2-generated structures of the MTBD and stalk of human DYNC2H1 (dynein-2 heavy chain) and DYH7 (inner arm axonemal dynein). Positions of residues equivalent to *Drosophila* S3372 are shown in red; alanines at residues equivalent to the cysteines at the base of CC2 in several other dyneins are also shown.

bioRxiv preprint doi: https://doi.org/10.1101/2023.08.03.551815; this version posted August 4, 2023. The copyright holder for this preprint (which was not certified by peer review) is the author/funder, who has granted bioRxiv a license to display the preprint in perpetuity. It is made available under aCC-BY 4.0 International license.

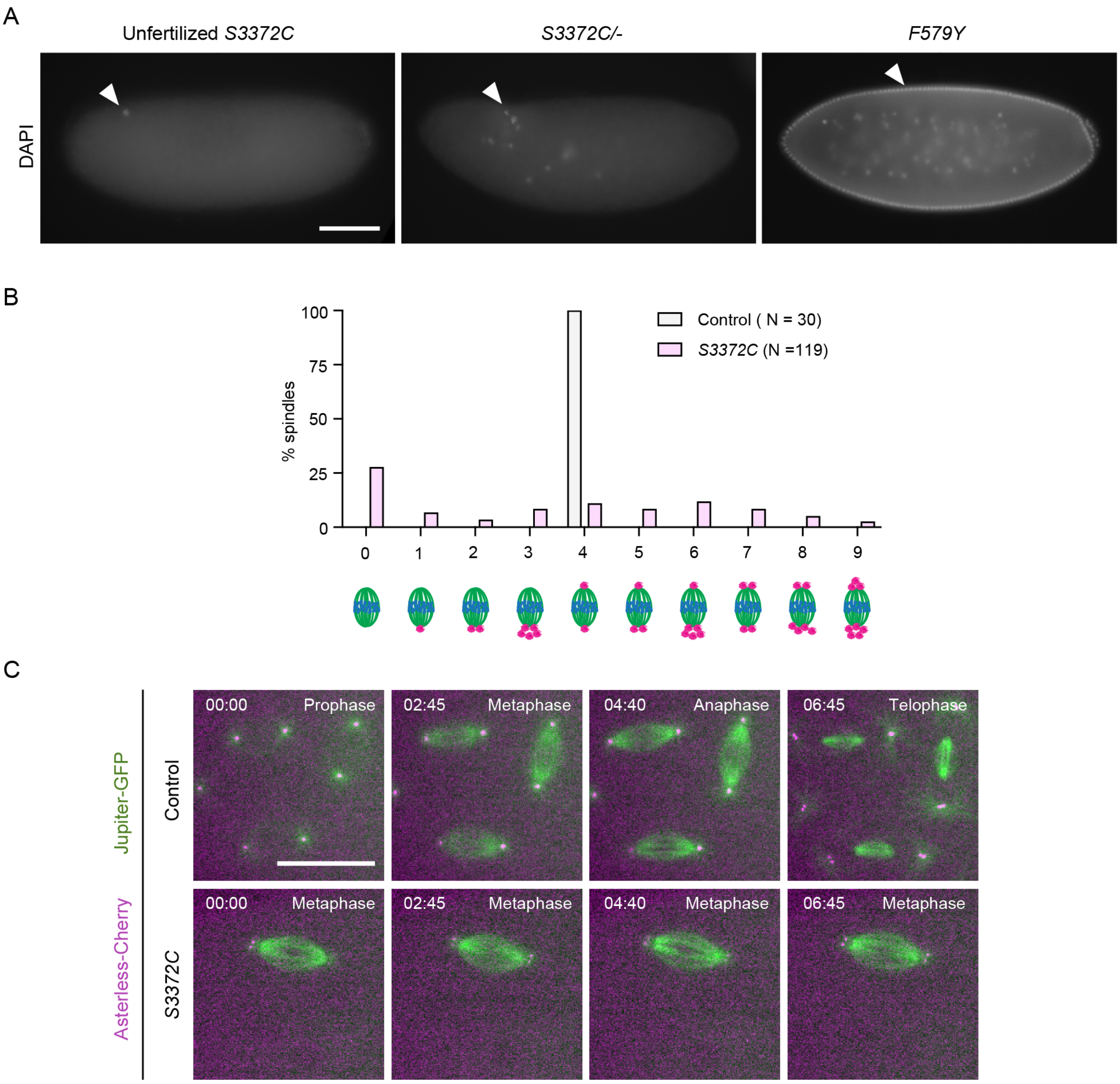

Figure S4. Supplementary data from coarse-grain analysis of S3372C mitotic phenotype. (A) Example wide-field images from a 2 to 4-h egg collection of fixed, DAPI-stained unfertilized eggs from virgin *S3372C* females, or embryos from mated *S3372C/-* or *F579Y* females (arrowheads show DNA staining). Images are representative of at least 150 embryos examined. (B) Categorization of mitotic spindle phenotypes in *S3372C* embryos based on centrosome number and arrangement. A range of mitotic stages were present in control embryos (*yw* strain), whereas >90% of mutant spindles were at metaphase; only those control and mutant embryos in metaphase were scored for this analysis. N is numbers of spindles scored (from 49 and 6 *S3372C* and control embryos, respectively). In both genotypes, no more than 5 randomly selected metaphase spindles were analyzed per embryo. (C) Example stills from time series (single focal plane) of control and *S3372C* embryos acquired during preblastoderm cycles. Jupiter-GFP and Asterless-Cherry label microtubules and centrosomes, respectively. Note abnormal presence of 2 centrosomes at each pole of the mutant spindle. In C, images were binned 2 x 2. Timestamps are min:s. Scale bars: A, 100 µm; B, 20 µm.

bioRxiv preprint doi: https://doi.org/10.1101/2023.08.03.551815; this version posted August 4, 2023. The copyright holder for this preprint (which was not certified by peer review) is the author/funder, who has granted bioRxiv a license to display the preprint in perpetuity. It is made available under aCC-BY 4.0 International license.

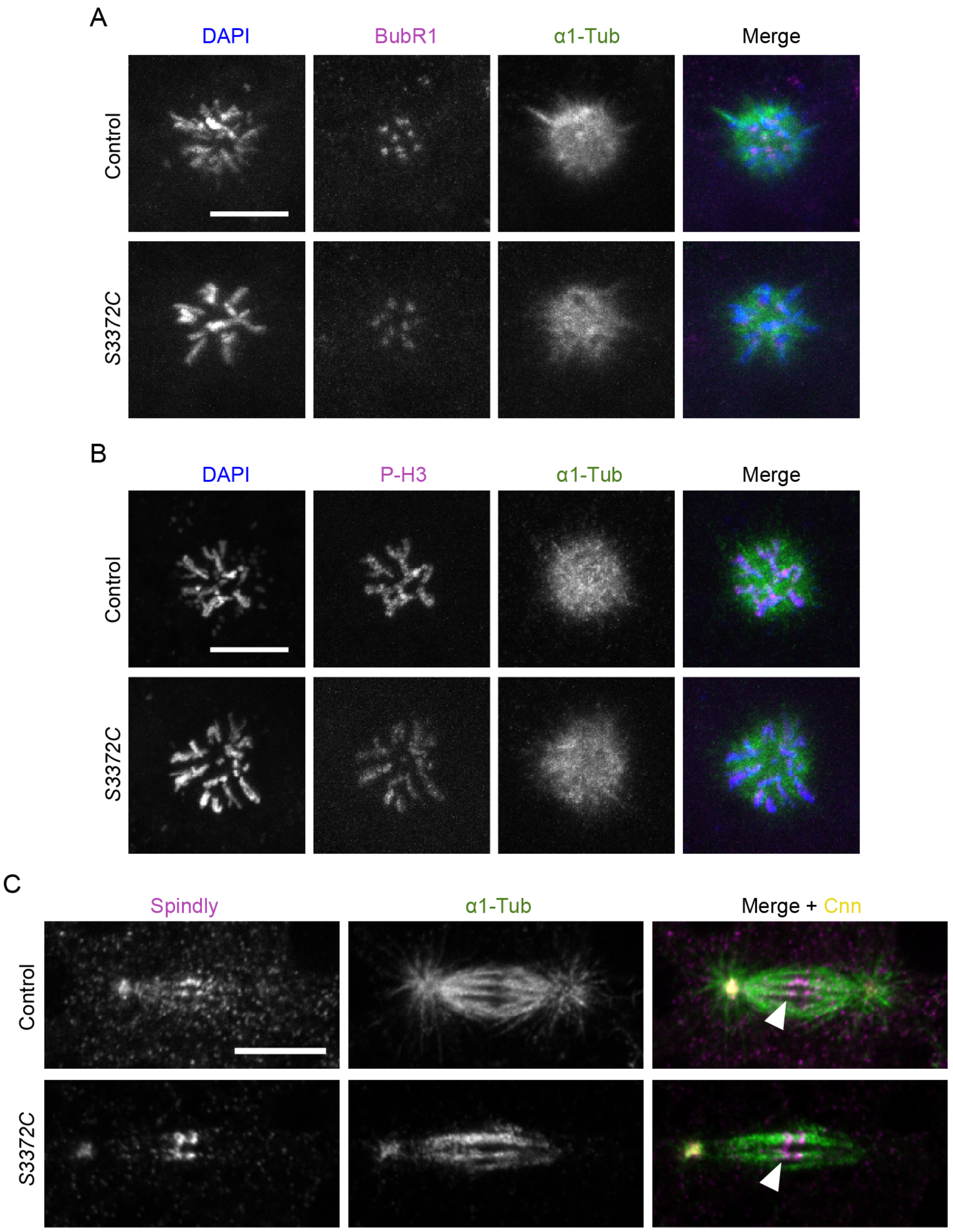

Figure S5. Supplementary data from fine-grained analysis of S3372C mitotic phenotype. (A, B) Example confocal images of polar bodies in fixed control and *S3372C* embryos stained with DAPI, as well as antibodies to (A) BubR1 and α1-Tubulin or (B) phospho-histone H3 (P-H3) and α1-Tubulin (Z-projections). (C) Example confocal images of mitotic spindles in control and *S3372C* embryos stained with antibodies to Spindly, α1-Tubulin and Centrosomin (Cnn), as well as DAPI. Arrowheads indicate example of close apposition of the ends of microtubule bundles and bright Spindly puncta at the kinetochore. Single focal planes were chosen to facilitate visualization of kinetochores, which resulted in only 1 centrosome being visible in each image. Timestamps are min:s. Scale bars in A – C, 10 μm. At least 50 embryos were examined per condition.

bioRxiv preprint doi: https://doi.org/10.1101/2023.08.03.551815; this version posted August 4, 2023. The copyright holder for this preprint (which was not certified by peer review) is the author/funder, who has granted bioRxiv a license to display the preprint in perpetuity. It is made available under aCC-BY 4.0 International license.

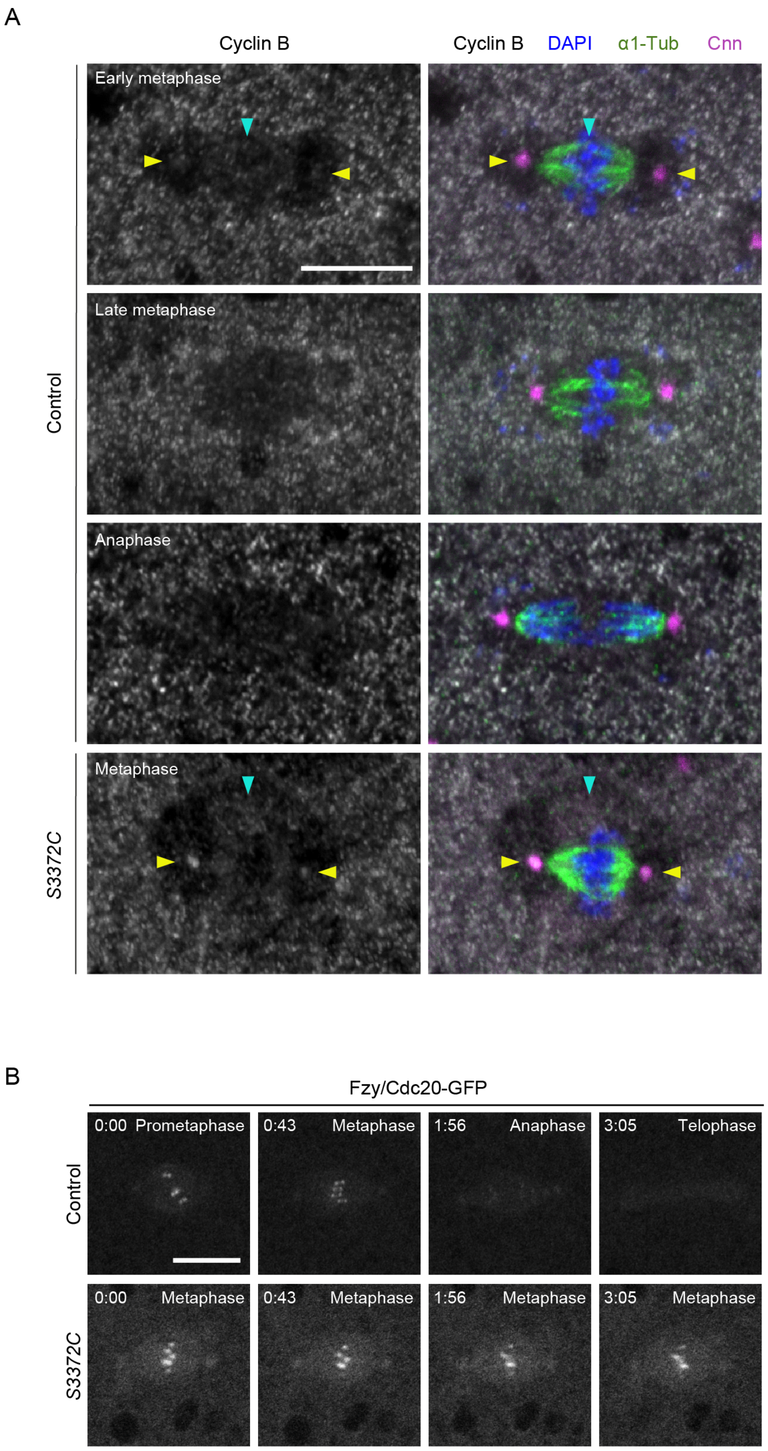

Figure S6. Analysis of Cyclin B and Fzy/Cdc20-GFP distribution on *S3372C* mutant spindles. (A) Example images of mitotic spindles during preblastoderm cycles in fixed control and *S3372C* embryos stained for the indicated antibodies, as well as with DAPI. Cyclin B shows weak accumulation in the vicinity of the spindle (blue arrowhead) and at centrosomes (yellow arrowheads) in early metaphase control embryos, which is lost by anaphase. In metaphase-arrested *S3372C* embryos, Cyclin B is detected in the vicinity of the spindle (blue arrowhead) and at centrosomes (yellow arrowheads). (B) Example stills of time series (single focal plane) of mitotic spindles acquired during preblastoderm cycles in live control and *S3372C* embryos expressing Fzy/Cdc20-GFP embryos. Fzy/Cdc20-GFP is localized to the metaphase plate in metaphase-arrested mutant spindles. Timestamps are min:s. Scale bars in A and B, 10 μm. At least 30 embryos were examined per condition.

bioRxiv preprint doi: https://doi.org/10.1101/2023.08.03.551815; this version posted August 4, 2023. The copyright holder for this preprint (which was not certified by peer review) is the author/funder, who has granted bioRxiv a license to display the preprint in perpetuity. It is made available under aCC-BY 4.0 International license.

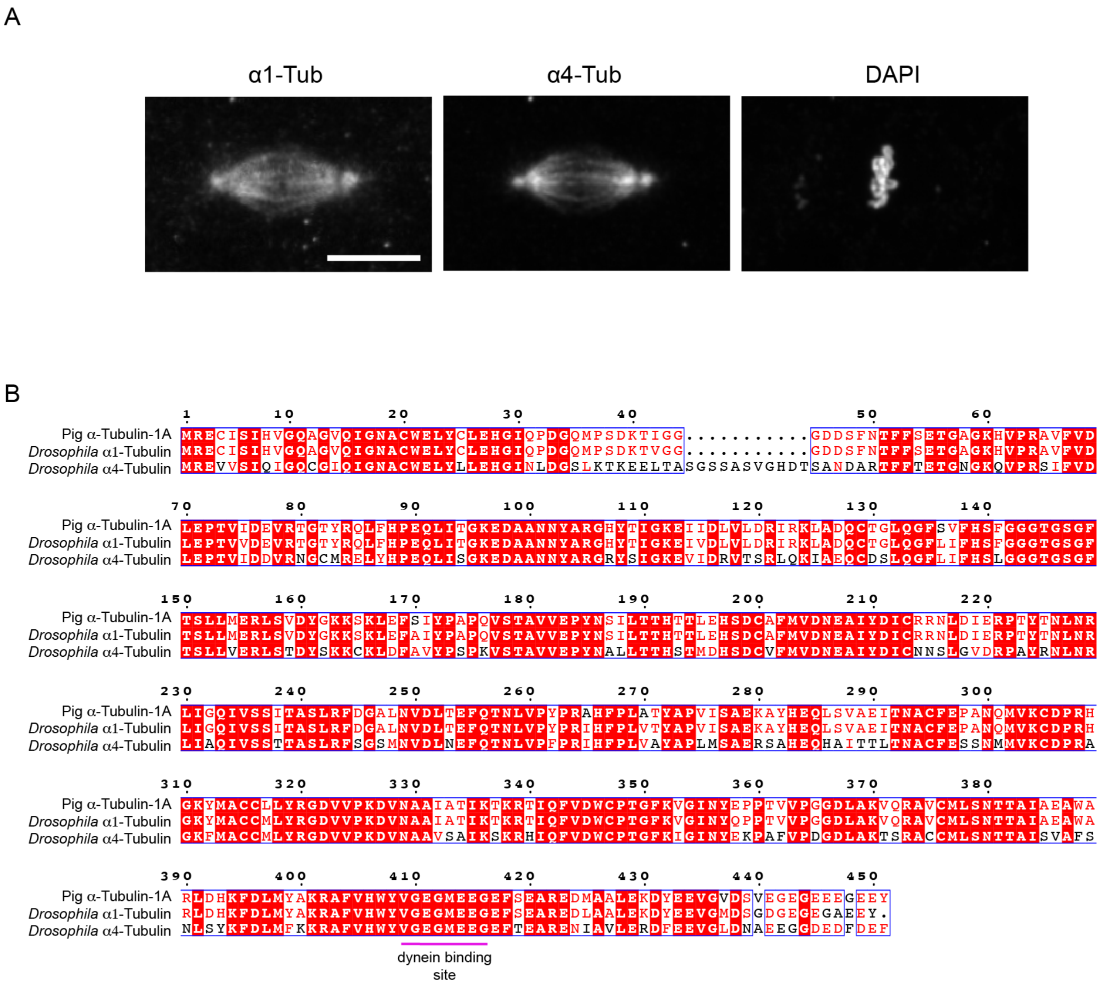

Figure S7. Assessing a potential α4-tubulin-specific effect of S3372C. (A) Example image of a metaphase spindle in wild-type embryo stained with antibodies to α1-tubulin and α4-tubulin, as well as DAPI. Scale bar: 10 μm. (B) Alignment of protein sequences of mammalian (pig) α-tubulin-1A, *Drosophila* α1-tubulin and *Drosophila* α4-tubulin. White letters on a red background indicate residues present in all sequences; red letters indicate residues present in ≥ 50% of sequences; blue boxes show regions with ≥ 50% conservation; magenta horizontal line, region contributing to dynein binding based on the mouse MTBD-microtubule structure (Lacey *et al*, 2019), which is identical in all 3 proteins. Uniprot accession numbers are: pig (*Sus scrofa*) α-tubulin-1A, P02550; *Drosophila melanogaster* α1-tubulin P06603; *Drosophila melanogaster* α4-tubulin P06606.

bioRxiv preprint doi: https://doi.org/10.1101/2023.08.03.551815; this version posted August 4, 2023. The copyright holder for this preprint (which was not certified by peer review) is the author/funder, who has granted bioRxiv a license to display the preprint in perpetuity. It is made available under aCC-BY 4.0 International license.

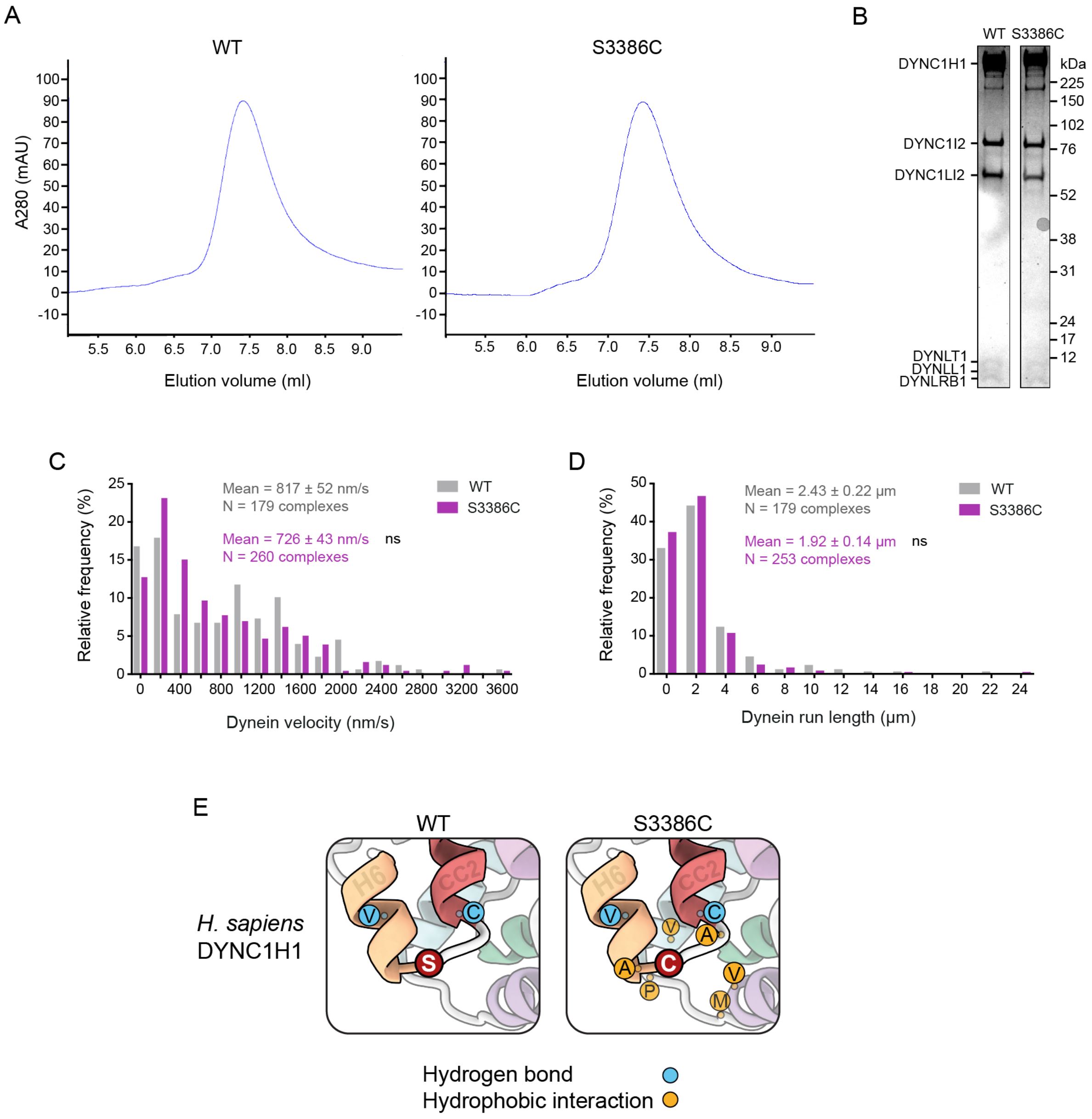

Figure S8. Supplementary data on *in vitro* and *in silico* analysis of S3386C human dynein. (A) Size-exclusion chromatography traces for wild-type (WT) and S3386C human dynein complexes, showing very similar profiles and lack of aggregation. (B) Cropped images from a Coomassie Blue-stained SDS-PAGE gel of pooled and concentrated fractions collected from the wild-type and S3386C mutant dynein peaks in A. (C, D) Velocity (C) and run length (D) frequency distributions for processive WT and S3386C mutant dynein complexes in the presence of dynactin and BICD2N in the assembly mix. Errors are S.E.M.. Evaluation of statistical significance was performed with a Mann-Whitney test. ns, not significant. (E) Zoom ins of regions of representative examples of MD-generated WT and S3386C mutant human dynein MTBD and partial stalk structures showing frequent hydrogen bonding interactions (blue circles) of S3386 and C3386 and new hydrophobic interactions of C3386 (gold circles); see Tables S1 – S3 for details of interacting residues.

bioRxiv preprint doi: https://doi.org/10.1101/2023.08.03.551815; this version posted August 4, 2023. The copyright holder for this preprint (which was not certified by peer review) is the author/funder, who has granted bioRxiv a license to display the preprint in perpetuity. It is made available under aCC-BY 4.0 International license.

SUPPLEMENTARY MOVIE LEGENDS

Supplementary movie 1. Live imaging of microtubules and chromatin in embryonic mitosis. Composite of example movies (generated with spinning disk confocal microscopy) of a single focal plane of control and *S3372C* preblastoderm cycle embryos. Jupiter-GFP (green) and His2Av-mRFP (magenta) label microtubules and chromatin, respectively. Images were collected at 0.2 frames s^{-1} with a playback rate of 10 frames s^{-1}. Timestamps are min:s. Scale bar, 10 µm. Related to Figure 5.

Supplementary movie 2. Live imaging of microtubules and centrosomes in embryonic mitosis. Composite of example movies (generated with spinning disk confocal microscopy) of a single focal plane of control and *S3372C* preblastoderm cycle embryos. Jupiter-GFP (green) and Asterless-Cherry (magenta) label microtubules and centrosomes, respectively. Images were collected at 0.2 frames s^{-1} with a playback rate of 15 frames s^{-1}. Images were binned 2 x 2. Timestamps are min:s. Scale bar, 10 µm. Related to Figure S4.

Supplementary movie 3. Live imaging of dynein and kinetochores in embryonic mitosis. Composites of example movies (generated with spinning disk confocal microscopy) of a single focal plane of control and *S3372C* preblastoderm cycle embryos. GFP-Dlic (green in top merged movies) and Spc25-RFP (magenta in top merged movies) label dynein complexes and kinetochores, respectively. Two loops of the movie are shown; the second loop pauses to show transient accumulation of GFP-Dlic at kinetochores (arrows). Images were collected at 0.2 frames s^{-1} with a playback rate of 10 frames s^{-1}. Timestamps are min:s. Scale bar, 10 µm. Related to Figure 6.

Supplementary movie 4. Live imaging of GFP-Rod streaming in embryonic mitosis. Composite of example movies (generated with spinning disk confocal microscopy) of a single focal plane of control and *S3372C* preblastoderm cycle embryos showing GFP-Rod dynamics. Streaming of faint GFP-Rod signals away from the kinetochores was analysis in kymographs. Images were collected at 0.5 frames s^{-1} with a playback rate of 10 frames s^{-1}. Timestamps are min:s. Scale bar, 20 µm. Related to Figure 7.

bioRxiv preprint doi: https://doi.org/10.1101/2023.08.03.551815; this version posted August 4, 2023. The copyright holder for this preprint (which was not certified by peer review) is the author/funder, who has granted bioRxiv a license to display the preprint in perpetuity. It is made available under aCC-BY 4.0 International license.

SUPPLEMENTARY TABLES

Table S1. Occurrence in simulations of hydrogen bond pairs involving S3386.

Donor (position)	Acceptor (position)	Occurrence (%) [1]
Ser3386 (loop)	Val3382 (helix 6)	61.33
Cys3389 (CC2)	Ser3386 (loop)	41.24

(1) 3.6 µs of all-atom MD simulations

bioRxiv preprint doi: https://doi.org/10.1101/2023.08.03.551815; this version posted August 4, 2023. The copyright holder for this preprint (which was not certified by peer review) is the author/funder, who has granted bioRxiv a license to display the preprint in perpetuity. It is made available under aCC-BY 4.0 International license.

Table S2. Occurrence in simulations of hydrogen bond pairs involving C3386.

Donor (position)	Acceptor (position)	Occurrence (%) [(1)]
Cys3386 (loop)	Val3382 (helix 6)	69.20
Cys3389 (CC2)	Cys3386 (loop)	32.32

(1) 3.6 μs of all-atom MD simulations

bioRxiv preprint doi: https://doi.org/10.1101/2023.08.03.551815; this version posted August 4, 2023. The copyright holder for this preprint (which was not certified by peer review) is the author/funder, who has granted bioRxiv a license to display the preprint in perpetuity. It is made available under aCC-BY 4.0 International license.

Table S3. Occurrence in simulations of hydrophobic interaction partners of C3386.

Partner (position)	Occurrence (%) [(1)]
Val3307 (helix 1)	99.94
Met3310 (loop between helices 1 and 2)	100
Pro3314 (loop between helices 1 and 2)	99.80
Val3317 (helix 2)	100
Val3382 (helix 6)	96.76
Ala3385 (loop between helix 6 and CC2)	100
Ala3388 (CC2)	100
Cys3389 (CC2)	100

(1) 3.6 μs of all-atom MD simulations

bioRxiv preprint doi: https://doi.org/10.1101/2023.08.03.551815; this version posted August 4, 2023. The copyright holder for this preprint (which was not certified by peer review) is the author/funder, who has granted bioRxiv a license to display the preprint in perpetuity. It is made available under aCC-BY 4.0 International license.

Table S4. Oligonucleotides used to create Dhc mutations in *Drosophila*.

***Drosophila* mutation**	**Human equivalent**	**gRNA cloning oligos (1)**	**Donor oligo (2)**
K129I (3)	*K129I*	f: gtcgCGCGGCCTGGTCGTGGAGG<br>r: aaacCCTCCACGACCAGGCCGCG	TCACTTCACCAACAGTCGGATGG<br>CATCTTTGGCCTGCATCAAGCGC<br>GGCCTGGTCGTGGA**A**GC**C**GACAt<br>cTCGATCCATTCGCAGCTGCGGC<br>TGATCAACTTCTCGGACGGATCT<br>CCCTACGAGACGCTG (s)
F579Y (4)	*F582Y*	f:<br>gtcgGAAAATGCGGAACATCTCGT<br>r:<br>aaacACGAGATGTTCCGCATTTTC	ATCAGCTGCGTCTGGTACTCCCG<br>GATAGCGCCACGGATGTGCGGAC<br>GCACGAAGAGCGCATTGAAACGC<br>GAGAAAATGCGG**T**ACATCTCGTT<br>GGCGTTCTTGGCTGTTCCCAGCT<br>GATCACGTAGATGAGCAGTTATG<br>CGAGTCTCCACGCGAT (as)
R1557Q	*R1567Q*	f: gtcgGAATTCGGAGCTGATGCTT<br>r: aaacAAGCATCAGCTCCGAATTC	ACCTTGGGCGATTTGGTCACCTT<br>CTTCATCAACCCAAGGAATTCGG<br>AGCTGATGCTTTG**A**AA**TT**GCGAA<br>GTCTCCACCGGAAGGAGCGTCTT<br>AATATCAGCGCTGCCCGAGAAGA<br>TTCCCTCCAA (as)
R1951C (5)	*R1962C*	f:<br>gtcgCGACGAGTTCAATCGACTGG<br>r:<br>aaacCCAGTCGATTGAACTCGTCG	GGCCGAATCTTTGTCGGTCTGTG<br>CCAGGTGGGCGCATGGGGCTGCT<br>TCGACGAGTTCAAT**T**G**TT**TGGAG<br>GAGCGTATGCTCTCCGCTTGCTC<br>ACAGCAAATCCAGACCATTCAGG<br>AGGCGCTGA (s)
K3226T	*K3241T*	f:<br>gtcgCATCTGCTTAAGCTTGGCGT<br>r:<br>aaacACGCCAAGCTTAAGCAGATG	CTGAATTTCCTGCGATTGAATCT<br>TCTTCTTCTCGGCCTCCTGCTGA<br>TCCTGGAACATCTG**TG**T**T**A**AT**TT<br>GGCGTTGGCAGCCTCGTTCTTGG<br>CCTGCAACTCCTGCTTCTTCACA<br>GCCAGCGACTTT (as)
R3370Q (6)	*R3384Q*	f: gtcgAAGGTTAATCGCGCCAGTA<br>r: aaacTACTGGCGCGATTAACCTT	GTATCAAAAAACAGCGATGATGT<br>TCGAGAGAAGATGAAGTCCAAAT<br>ATCTGAGCAATCCGGACTATAAC<br>TTCGAGAAGGTTAATC**AA**GC**G**AG<br>TATGGCGTGTGGTCCTATGGTAA<br>AATGGGCCATTG (s) (7)<br><br>CGATGATGTTCGAGAGAAGATGA<br>AGTCCAAATATCTGAGCAATCCG<br>GACTATAACTTCGAGAAGGTTAA<br>TC**AA**GC**G**AGTATGGCGTGTGGTC<br>CTATGGTAAAATGGGCCATTGCT<br>CAGGTAAGATTTATATCGAATTG<br>ATCGTGAATAG (s)

bioRxiv preprint doi: https://doi.org/10.1101/2023.08.03.551815; this version posted August 4, 2023. The copyright holder for this preprint (which was not certified by peer review) is the author/funder, who has granted bioRxiv a license to display the preprint in perpetuity. It is made available under aCC-BY 4.0 International license.

S3372C+ C3375S	*S3386C+ C3389S*	f: gtcgAAGGTTAATCGCGCCAGTA<br>r: aaacTACTGGCGCGATTAACCTT	GTATCAAAAAACAGCGATGATGT<br>TCGAGAGAAGATGAAGTCCAAAT<br>ATCTGAGCAATCCGGACTATAAC<br>TTCGAGAAGGTTAATCGCGCC**T**G<br>TATGGCG**A**GTGGTCCTATGGTAA<br>AATGGGCCATTG (s)
C3375S	*C3389S*	f: gtcgAAGGTTAATCGCGCCAGTA<br>r: aaacTACTGGCGCGATTAACCTT	GTATCAAAAAACAGCGATGATGT<br>TCGAGAGAAGATGAAGTCCAAAT<br>ATCTGAGCAATCCGGACTATAAC<br>TTCGAGAAGGTTAATCGCGC**T**AG<br>**C**ATGGCG**A**GTGGTCCTATGGTAA<br>AATGGGCCATTG (s)
H3808P (8)	*H3822P*	f:gtcgTCTTTAGCGAGTACTGGTAG<br>r:aaacCTACCAGTACTCGCTAAAGA	GGTTATTATACAGCACCGTGGAG<br>AAGATATCGAGGAACATCTTTAG<br>CGAGTACTGGTAGAG**A**AAG**G**GCA<br>CCTGGTTCAGACTGTCCATGGTG<br>AAGTAGATGTTGCTGCACGCCAC<br>AGAAAGTGGGA (as)

(1) f, forward; r, reverse. Sequences corresponding to target sequence are upper case; sequences used for cloning of the annealed oligo pairs into the gRNA plasmid pCFD3 are lower case
(2) Donors correspond to either the sense (s) or antisense (as) strand, as indicated. Positions that differ from the equivalent region of the target genome are shown in bold. In cases where a disease mutation does not interfere with recutting by the Cas9/gRNA complex, synonymous mutations in the seed sequence or protospacer adjacent motif were also introduced
(3) The *Dhc*^{null} allele was also recovered in this experiment
(4) Corresponds to the mouse *Loa* mutation, which is F580Y in the orthologous Dync1h1 protein
(5) The *L1952K* allele was also recovered in this experiment
(6) The *S3372C* allele was also recovered in this experiment. The same gRNA plasmid was used to make the *S3372C + C3375S* and *C3375S* alleles
(7) 2 different donors, which are shifted with respect to each other in the 5′ to 3′ direction, were injected into separate batches of embryos. Each donor led to recovery of both *R3370Q* and *S3372C* alleles
(8) The *ΔL3810+Y3811* and *Y3811F* alleles were also recovered in this experiment

bioRxiv preprint doi: https://doi.org/10.1101/2023.08.03.551815; this version posted August 4, 2023. The copyright holder for this preprint (which was not certified by peer review) is the author/funder, who has granted bioRxiv a license to display the preprint in perpetuity. It is made available under aCC-BY 4.0 International license.

Table S5. Primary antibodies used in this study.

Target	Host	Catalog number / Source [(1)]	Dilution for IF / IB [(2)]
α-Tubulin	Mouse	T6199 (clone DM1A) / Sigma Aldrich	1:500 / NA
α-Tubulin	Rabbit	EP1332Y / Abcam	NA / 1:2000
α4-Tubulin	Rabbit	NA / Fahmy *et al*, 2014	1:400 / NA
BubR1	Rabbit	NA / Logarinho *et al*, 2004	1:1000 / NA
Centrosomin	Sheep	NA / Lucas & Raff, 2007	1:500 / NA
Cyclin B	Rabbit	NA / Whitfield *et al*, 1990	1:500 / NA
Cysteine string protein	Mouse	ab49 / Developmental Studies Hybridoma Bank (DSHB)	1:250 / NA
Dynein heavy chain	Mouse	2C11-2 / DSHB	NA / 1:1000
Phospho-histone H3	Rabbit	9701 / Cell Signalling	1:200 / NA
Spindly	Rabbit	NA / Griffis *et al*, 2007	1:1000 / NA
Synaptotagmin	Rabbit	NA / West *et al*, 2015	1:1000 / NA

(1) NA, not applicable
(2) IF, immunofluorescence; IB, immunoblotting; NA, not applicable

bioRxiv preprint doi: https://doi.org/10.1101/2023.08.03.551815; this version posted August 4, 2023. The copyright holder for this preprint (which was not certified by peer review) is the author/funder, who has granted bioRxiv a license to display the preprint in perpetuity. It is made available under aCC-BY 4.0 International license.

Table S6. Secondary antibodies used in this study.

Target	Host	Catalog number / Source [(1)]	Dilution for IF / IB [(2)]
Donkey anti-mouse IgG (H+L), Alexa488		A-21202 / ThermoFisher Scientific	1:500 / NA
Donkey anti-Rabbit IgG (H+L), Alexa555		A-31572 / ThermoFisher Scientific	1:500 / NA
Donkey anti-Sheep IgG (H+L), Alexa647		A-21448 / ThermoFisher Scientific	1:500 / NA
Ms m-IgGκ BP-HRP		sc-516102 / Santa Cruz	NA / 1:10,000
anti-Rabbit IgG, HRP-linked whole antibody		NA934V / GE Healthcare	NA / 1:10,000

(1) NA, not applicable
(2) IF, immunofluorescence; IB, immunoblotting; NA, not applicable
